# Aterian shell beads from the coastal site of El Mnasra Cave (Rabat-Témara, Morocco): Specificities of the north African MSA personal ornaments

**DOI:** 10.1371/journal.pone.0338785

**Published:** 2026-03-25

**Authors:** Emilie Campmas, Matthieu Lebon, Catherine Dupont, Manon Bondetti, Eslem Ben Arous, Arnaud Lenoble, Driss Chahid, Pierre Lozouet, Ludovic Bellot-Gurlet, Mohamed Abdeljalil El Hajraoui, Roland Nespoulet

**Affiliations:** 1 TRACES, UMR 5608-CNRS, University of Toulouse Jean Jaurès, Toulouse, France; 2 Histoire Naturelle des Humanités Préhistoriques -UMR 7194, Muséum National d’Histoire Naturelle, CNRS, University Perpignan Via Domitia, Musée de l’Homme, Paris, France; 3 CReAAH, UMR 6566-CNRS, Rennes University, Campus Beaulieu, Rennes, France; 4 Department of Archaeology/BioArch, University of York, York, United Kingdom; 5 Max Planck Institute of Geoanthropology, Human Palaeosystems Group (MPI-GEA), Jena, Germany; 6 PACEA-UMR 5199, Université de Bordeaux, CNRS, MCC, Bordeaux, France; 7 Université Cadi Ayyad Ecole Normale Supérieure de Marrakech, Laboratoire de Recherche Interdisciplinaire en Bioressources, Environnement et Matériaux (LIRBEM), Marrakech, Maroc; 8 Muséum National d’Histoire Naturelle, Direction des Collections, Paris, France; 9 Sorbonne Université, CNRS, de la Molécule aux Nano-objets: Réactivité, Interactions et Spectroscopies, MONARIS, Paris, France; 10 Institut National des Sciences de l’Archéologie et du Patrimoine, Rabat, Morocco; Sapienza University of Rome: Universita degli Studi di Roma La Sapienza, ITALY

## Abstract

The use of Nassariidea shells as personal ornaments is attested to an increasing number of Middle Stone Age (MSA) archaeological sites in northern and southern Africa. The chronological extent of this behavior is constantly moving back in time; currently, the oldest evidence has been identified at the Bizmoune cave site in Morocco back to the MIS 6. Although these evidences make it possible to refine the spatial and temporal distribution of this behavior, shell beads remain rare in Middle Stone Age assemblages and are generally composed of several beads, or at best dozens, for each of these sites. This restricts our understanding of the behaviors specifically related to the collection, selection and preparation phases of shells, and potentially limits our understanding of their use. In this article, we studied shell beads from MSA layer US 8 from the coastal archaeological site of El Mnasra Cave (Rabat-Témara, Morocco). This collection corresponds to the largest MSA shell bead assemblage in Africa (272 *Tritia* cf*. gibbosula, 6 Tritia corniculum* and *3 Columbella rustica* in US 8 with 154 of them showing smoothing of the perforation edge, facet of abrasion, or traces of pigment). The shell bead assemblage of El Mnasra presents features previously observed at other MSA sites, connecting it to a North African cultural context; however, the size of the El Mnasra shell bead assemblage, and the presence of shell sources near the site, allows us to identify specific features that could be related to particular modes of use as ornaments. These specific features include the prevalence of un-perforated shells, some of which show use-wear, that could have been fixed on items without having been perforated. These results provide new insights into the wide range of variants and originalities of shell bead uses over a relatively “short” chronological phase, between 115 and 94 ka and can be correlated with the multistep evolutionary scenario proposed for South Africa. The archaeological documentation presented here shows that El Mnasra Cave provides a significant contribution to the study of culturing the Palaeolithic body in North Africa.

## Introduction

Personal ornaments correspond to standardized items that shared symbolic meaning among a particular group or other groups of the same culture [1,2]. The discovery of ancient ornaments in Middle Stone Age (MSA) archaeological contexts has therefore focused the scientific community’s attention on the ongoing debate concerning the appearance of a “cultural modernity” [3–9]. They are regarded as one of the most important archaeological artifacts evidencing the emergence of more complex human social interactions and a culturalization of the human body [10,11]. Nassariidae shells used as ornaments are commonly found in Middle Stone Age (MSA) archaeological contexts in the Levantine area and Southern and Northern Africa sites, dated between 100 and 80 ka, and even earlier in the latter. In fact, the oldest evidence of shell ornaments dating back to the Middle Pleistocene was recently found at the Bizmoune Cave site in Morocco [12]. From these earliest manifestations, the use of perforated shell beads shows a degree of standardization with similar modes of use. With the exception of Qafzeh Cave (Israel), which yielded perforated bivalves of *Glycymeris* [13], all the species used throughout the African continent, are also similar in shape and are phylogenetically close [10]: *Nassarius kraussinarius* in Southern Africa (Blombos); *Tritia gibbosula* and *Nassarius circumcintus* in Northern Africa (Bizmoune, Contrebandiers, El Mnasra, Taforalt, Ifri n’Ammar, Rhafas, Oued Djebanna) and the Levantine area (Skhul) [14–22]. More recently, the presence of unperforated shells in 100 ka levels at Blombos Cave led d'Errico et al. [10] to suggest that these shells were collected and used as cultural items in the form of amulets or similar objects. For these authors, the use of un-perforated shells as cultural objects falls within a ten-step evolutionary scenario of the culturalization of the human body. The use of un-perforated shells would therefore precede a generalization and standardization of the use of shell as ornaments in beadwork configuration, with an increasing complexity of the bead's nature (naturally or artificially perforated shell, ochre or heating color modifications, association of different species), as well as types and modalities of bead arrangements.

In this respect, the MSA site of El Msara Cave (Témara – Morroco) is a key site for understanding the early stages in the use of ornaments. Excavations from 2005 to 2014 using rigorous scientific protocols has yielded a larger number of Nassariidae shell beads [21], especially in stratigraphic unit 8 (US 8), dated from 115−94–102−74 ka with 95% probability [23,24]. These dates suggest that these shell beads are among the oldest evidence of ornamentation in Africa, including those previously discovered in the Moroccan site (See for example Bizmoune (> 147 ka; [12]), Contrebandiers (115−95 ka; [19]), El Harhoura 2 (92 ka; [21,25]) and Taforalt (Unit E, ~ 80 ka by TL; [15])). In particular, the number of specimens from El Mnasra corresponds to the largest and most diversified assemblage (N = 322) for the entire MSA record in Africa [9,26]. In addition to perforated shell beads, the presence of unperforated shells in the El Mnasra archaeological assemblage could support the hypothesis of an early phase in the use of shells as a cultural element.

This paper aims to complement the previous study on the ornaments of El Mnasra Cave [21] and provide new taphonomic data presenting the shell origins and the hypotheses of their use. More specifically, we present the results for El Mnasra stratigraphic unit 8 (US 8) shells identified as ornaments, in comparison with similar shells from a paleontological thanatocoenosis near El Mnasra cave. Several aspects have been considered, such as spatial distribution in the cave, and the species, size, morphology, surface conservation, perforations, use-wear, heating evidence and pigment residues of the shells. The origin, human selection, gathering zone locations and uses are discussed for all species.

## Regional and site setting

**Fig 1:**
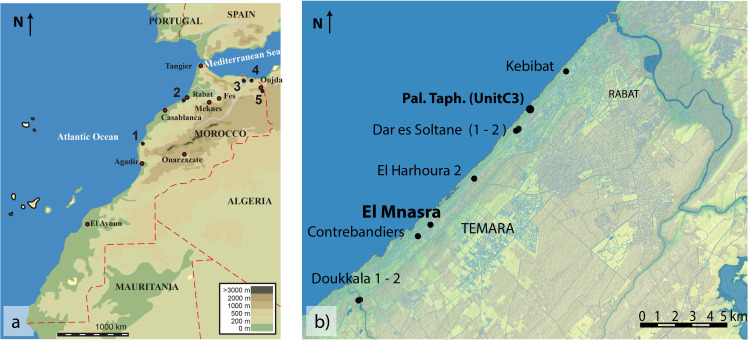
Location of the El Mnasra Cave and Moroccan site mentioned in the text. a) Location of the Témara-Rabat region and the other Northwestern African sites that have yielded Aterian shell ornaments [1: Bizmoune, 2: Témara-Rabat sites, 3: Ifri n’Ammar, 4: Taforalt, 5: Rhafas]. b) Location of the El Mnasra Cave and details of the archaeological sites of the Rabat-Témara region (Dar es Soltane 1, El Harhoura 2, Contrebandiers, Doukkala 1 and 2); Location of the paleontological thanatocoenosis of Dar es Soltane (Unit C3); Contains information from OpenStreetMap and OpenStreetMap Foundation, which is made available under the Open Database License.

The archaeological cave of El Mnasra (33°55’ 40.9”N, 6° 57’ 13.3” W) is located in the Témara region of the Atlantic Coast, a few kilometers south of Rabat. This area presents an exceptional concentration of archaeological sites with several coastal caves containing similar sedimentary deposits yielding MSA assemblages: Doukkala 1 and 2 Caves, Contrebandiers Cave, El Mnasra Cave, El Harhoura 1 and 2 Caves, and Dar es Soltane 1 and 2 Caves (Fig 1). These archaeological assemblages have yielded evidence of the significant use of various marine resources, especially mollusk shells such as Mytilidae, Patellidae, Trochidae, Muricidae and Nassariidae [16,19,21,22,25,27–30].

**Fig 2:**
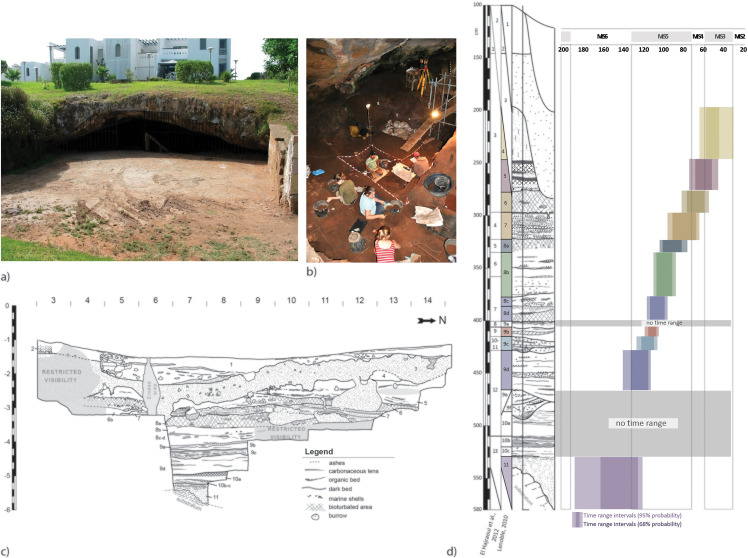
El Mnasra Cave. a) Photograph of the El Mnasra Cave entrance and b) During excavation of US 8 in 2013 [Photograph R. Nespoulet]. c) Lithostratigraphy established by A. Lenoble, d) Synthesis of the Middle Stone Age chronology from El Mnasra cave published by Ben Arous, Philippe [24], according to archaeological stratigraphy [35] and lithostratigraphy (Lenoble [Unpublished]).

The El Mnasra Cave (Fig 2a, b) was discovered by J. Roche in 1956, with several excavation campaigns being conducted between 1990 and 2002 by M.A. El Hajraoui. Since 2005, this cave has been included in the archaeological program of El Harhoura 2-Témara (directed by R. Nespoulet and M.A. El Hajraoui). This site is located 500 m from the actual shoreline at 14 m above the current sea level. The cave opens onto a fossil cliff, which was carved into the calcarenite during the high sea level phases of the last interglacial, ca. 130‒125 ka [23,31–33].The cave’s dimensions are 22 m x 17 m x 6m and the excavation area covers a maximum surface of 28 m². An oculus is present in the roof at the rear part of the cave. The stratigraphy of El Mnasra is complex (Fig 2c), due to variable geometries of sediment layers as well as recent perturbations corresponding to 1) a modern digging and trampling area perhaps resulting from the work of a gardener (probably to collect fertile earth), or water runoff leading to the collapse of archaeological sections in the excavation squares (~2 m^2^) and 2) large burrows, in a large part attributable to honey badgers (*Mellivora capensis*) ‒ the skeleton of one specimen, still partially articulated, was identified in one of the burrows [34].

The stratigraphy was established by M.A. El Hajraoui and A. Debénath [35]. From top to bottom, 13 archaeological layers are described: a disturbed layer (Level 1), a Neolithic layer (Level 2), 8 MSA layers (Levels 3–10) and 3 archaeologically sterile layers (Levels 11–13). This stratigraphy was revised by A. Lenoble (Lenoble, 2010, 2011 [Unpublished]) who defined a new litho-stratigraphy based on 11 stratigraphic units (“US”) grouped into 6 “Members” [24]. From the top to the base:

- Member 1: USs 1 and 2 – modern elements mixed with Holocene deposits (US 1) and *in situ* Holocene deposits (US 2)
- Member 2: US 3 – Dark reddish-brown sand resulting from bioturbation induced by burrowing animals that reworked USs 4 and 5 and, to a lesser extent, the underlying USs 6–8.
- Member 3: USs 4 and 5 – Red-brown fine clayey sand (US 4); Reddish-brown to light reddish-brown fine clayey sand interspersed by several centimeter-thick beds of black organic clayey sand (US 5) corresponding to continental sedimentation during the Pleniglacial period (“Soltanian”).
- Member 4: USs 6 and 7. Brown to dark grey fine sand, massive and rich in archaeological artifacts, lenses of anthropogenic sediments (ashes, charcoal), and mollusk shells (US 6); Bedded sandy loam cemented on top by carbonated beds interpreted as dominated by ashes and/or hearth cleaning. Some synsedimentary micro-bioturbations were observed in these units.
- Member 5: USs 8–10 – Bedded sandy-clay deposit including archaeological artifacts more or less intercalated with ashy or charcoal-rich lenses, while large cut-and-fill structures (e.g., US 9e/9f) point to high energy runoff events.
- Member 6: US 11 – carbonated sands with rare marine shell fragments corresponding to a storm coastal deposit due to the high sea level of the MIS 5.5.

This study focuses on stratigraphic unit 8 (US 8) which is the richest of all the MSA levels from El Mnasra and was excavated over 25 m^2^. Stratigraphic unit 8 correspond to levels 5, 6 and 7 of the archaeo-stratigraphy. These layers were dated by OSL (optically stimulated luminescence) to approximately ~117−105 ka [23,36]. New paleodosimetric dates were recently obtained by OSL and combined US-ESR (electron spin resonance combined to U-series dating), and a chronostratigraphic Bayesian model (Fig 2d) was proposed for the MSA sequence of El Mnasra [24]. This model is coherent with the paleo-environmental reconstitution based on large and small vertebrates [24,25,27,30] and lithostratigraphy analysis. This study provides a period estimation between 139 and 111 ka for the first occupations attested to in US 9d, and placed over the US 8 from 115−94 to 102−74 ka with 95% probability. The MSA industries of El Mnasra are characteristic of the Aterian, with the presence of tanged tools, Levallois/micro-Levallois debitage (flakes and cores) and side-scrapers [37]. El Mnasra also yielded evidence of bone tools [38] and pigment use in an Aterian MSA context in US 8 [39]. Hearths were found in US 8b and 8a (levels 5 and 6), some of them delimited by rock structures [40], or characterized by the presence of intense combustion areas, clearly delimited by limestone pavements in US 8b and 9a (levels 6 and 8). Human remains were found in US 8b and 8a [41].

A total of 1130 lithic pieces, including many highly fragmented pieces (N = 794), were discovered. Flakes (N = 871) were the most numerous, with 62 blades and bladelets. Retouched pieces were scarce (N = 84), and the tools (N = 109) correspond to a lithic reduction rate of 12%. The tools assemblage is similar to what is known from the Aterian in this region of Morocco, with the presence of pedunculated pieces (N = 18), scrapers (N = 33) and macro-tools (choppers and chopping-tools, N = 14). The presence of Levallois micro-nuclei (N = 6) was also noted. The raw materials are diversified (flint, limestone, quartzite, magmatic rock and quartz), mainly local and rarely of good quality [42]. They testify to fragmented *chaînes opératoire*. The lithic pieces attest to diversified activities by mobile groups in a nearby environment [30]. There are a total of 13 bone tools, some of which are ochre-colored, consisting mainly of points and lissoirs obtained by abrasion on long vertebrate bones [30,43]. More than 50 hematite pieces (“ochre”), plus a concave surface pebble used as a grinding stone retaining traces of hematite, were found in US 8 [44]. Lastly, eight hearth structures were found in US 8, one of which was associated with a 1 m² stone pavement [40].

The micro-faunal and paleo-environmental study of El Mnasra is ongoing (Stoetzel, analysis in progress); however, US 8 has thus far yielded remains attributed to the rodent *Arvicanthis* sp., occurring today in sub-Saharan savannas and the Nile Valley. Its presence at El Mnasra during the Late Pleistocene indicates a savanna-type environment and the probable existence of migration routes from the south and/or the east during wetter periods [27,34,45]. This stratigraphic unit has also yielded a large range of meso- and macro-faunal remains, mainly resulting from human occupation [25,29,34,38]. The archaeological artifact density is relatively low in US 8 and a low number of animals were exploited.

In addition to these terrestrial faunal remains, a large number of marine mollusk shells were unearthed during the excavation of US 8. They come from various species of Patellidae, Mytilidae, Trochidae and Muricidae [29,30]. Although it is not possible at this stage of the study to determine precise number of remains, minimum number of individuals and weight associated, limpets and mussels dominate the assemblage and their density around 264 N/m^3^ is of the same order of magnitude as faunal remains (312 N/m^3^) or lithic industry (208 N/m^3^) [29]. We can emphasise the fact that US 8 was deposited in a period subsequent to the high sea level of the MIS 5.5 stage.

Geological and geomorphological studies conducted by several members of our team have made it possible to refine this sea level [32,46]. The coastal region of Témara features a series of elongated coastal ridges that are subparallel to the current coastline. Morpho-litho-stratigraphic and geochronological studies (OSL dating) conducted on the most recent coastal ridge, very close to the current shoreline, revealed that it corresponds to a consolidated dune ridge, dated to isotopic stage 5c (104 ± 8–94 ± 7 ka), i.e., contemporary with US 8. The sea level was then lower than the current level.

This period, marked by a lower sea level, led to sediment deposition through the remobilisation by runoff of coastal sediments, mainly aeolianites, in a secondary position [24].

The distance between El Mnasra Cave and the shoreline, and the absence of marine contribution to the sedimentary accumulation of the US 8 attested that the shells found in US 8 can only be attributed to human activity. Indeed, research conducted on several MSA sites in the Témara region (Contrebandiers, El Harhoura 2, Dar es Soltane 1) have highlighted the integration of marine resources into the diet of the Aterian populations [22,28,29]. These food refuses are not the only marine shell remains present in US 8, since shells of smaller species with low nutritional interest and bearing perforations, use-wear, or pigments were also found during excavations.

All these features suggest that US 8 corresponds to brief occupations by mobile groups of Aterian hunter-gatherers, attesting to multiple activities directly linked to the exploitation of marine and terrestrial resources.

## Materials and methods

### Archaeological material

Numerous Nassariidae shells were unearthed during the excavation of the US 8. The recurrence of the use of Nassariidae species as body ornaments during the MSA and LSA in Africa, and presence of evidences of use as shell beads classically observed for other archaeological assemblages (perforations, use-wear and pigments) on a significant proportion of Nassariidea from US 8, led to particular attention being paid to these remains during the excavation. All shell remains of species showing use-wear (*Tritia* cf. *gibbosula, Tritia corniculum; Columbella rustica)*, or corresponding to species with similar size or morphology (*Ocenebra* sp.*)*, have been carefully examined for evidence of their use as ornaments.

**Fig 3:**
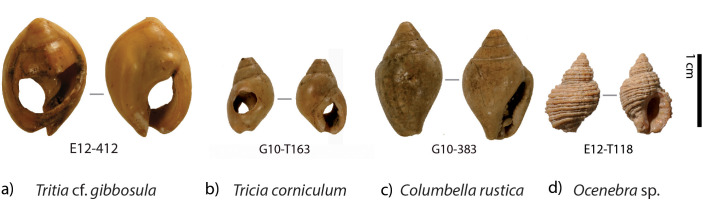
Examples of species proposed as potential ornaments from the US 8 of the El Mnasra excavation a) *Tritia* cf. *gibbosula*, b) *Tritia corniculum*, c) *Columbella rustica,* d) *Ocenebra* sp.

**Fig 4:**
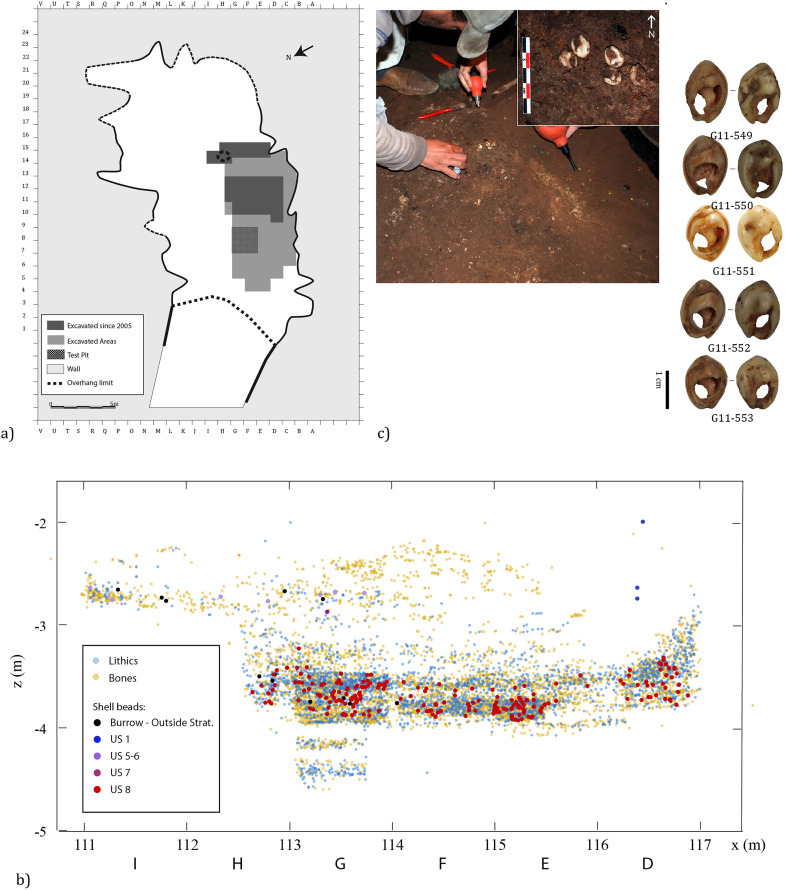
El Mnasra cave excavations. a) Spatial distribution of excavated areas. b) Stratigraphical distribution (X-Z) of shell ornaments according to the lithostratigraphy (Lenoble, 2010 [Unpublished]). c) Concentration of five *Tritia* cf*. gibbosula* discovered in the 2010 excavation campaign in square G11 of US 8 at El Mnasra cave (Photographer: R. Nespoulet) and pictures of these shells (photographers: M. Bondetti, B. Leprêtre, E. Lesvignes and M. Lebon).

All the shells suspected of being ornaments were excavated between 2005 and 2014 by the “El Harhoura-Témara” archaeological team, directed by M.A. El Hajraoui and R. Nespoulet. The material studied included specimens previously presented in a preliminary study by El Hajraoui in 2012 (N = 179) and collected during the following excavation campaigns. At El Mnasra, 322 mollusk shells attributed to *Tritia* cf. *gibbosula, Tritia corniculum, Columbella rustica,* and *Ocenebra* sp*.* were identified (Fig 3). These shells were discovered in several stratigraphic units (US), though the majority (N = 284) came from US 8 (Table 1 and Fig 4) which is the subject of this study (see the complete information for these specimens in S1 Table). Twenty-six specimens, identified as coming from reworked sediments, especially burrows (out of stratigraphical context) were not studied. The spatial distribution of shell ornaments was realized with Qgis3 software and modified with Illustrator software.

**Table 1:** Number of shells attributed to *Tritia* cf. *gibbosula, Tritia corniculum, Columbella rustica,* and *Ocenebra* sp. during excavation campaigns from 2005 to 2014 by stratigraphic unit (US). The shells out of stratigraphical context were found in the burrows.

				*Tritia* cf. *gibbosula*		*Tritia corniculum*		*Columbella rustica*		*Ocenebra* sp.	
Stratigraphic Units	Age [24]	N	%N	N	%N	N	%N	N	%N	N	%N
US-1	Neolithic	3	0.9	3	100						
US-5‒6	80−44 ka	7	2.2	7	100						
US-7	94−62 ka	2	0.6	2	100						
US-8	115−74 ka	284	88.2	272	95.8	6	2.1	3	1.1	3	1.1
Out of stratigraphical context	–	26	8.1	26	100						
TOTAL		322	100								

This study focuses purely on US 8 because it yielded an exceptionally high concentration of shells attributed as ornaments during excavation (burrows and outside stratigraphy excluded (“OS”); N = 284). No particular spatial distribution was observed; however, some clear associations of specimens were identified during excavation, e.g., at z = −3.5 m in area G11 (Fig 4c).

### Paleontological material

Fossil Nassariidae shells were collected from a natural coastal thanatocoenosis (“Dar es Soltane - Unit C-3 thanatocoenosis”, DeS-C3), 8.5 km north of El Mnasra (33°59’10.30"N, 6°53’31.97"W), near the archaeological site of Dar es Soltane 1 (Fig 1b).

The Atlantic coastline of the Témara area presents a succession of palaeobeach formations subparallel to the present shoreline. These parallel coastal ridges were formed during the Pleistocene following sea-level fluctuations and tectonic activity.

The most recent coastal ridges evidence marine deposits containing large amounts of malacofauna remains. This is especially the case for the marine calcarenite layer studied by Chakroun, Chahid [47], which yielded a thanatocoenosis composed of, among others, *Columbella rustica*, *Patella vulgata, Cardium tuberculatum, Stramonita haemastoma*, *Purpura lapillus*, *Cymatium cutaceum*, *Natica hebraea*, and several species of Nassariinae (formally named *Nassarius reticulatus*, *Nassarius circumcinctus*, *Nassarius gibbosulus*). Other sedimentary units corresponding to supratidal deposits, characterized by the color red (Unit C-3; [46]), presented a malacofauna thanatocoenosis composed of a mixture of continental (*Helix vermiculata, Helix lapicida, Rumina decollata*) and marine species (*Nassarius reticulatus*, *Nassarius circumcinctus*, *Columbella rustica, Cardium* sp.*, Ostrea* sp.*, etc).* In some locations, these red deposits partially consolidated presented a malacofauna dominated by Nassariinea, such as near Dar es Soltane (“Unit-C3 thanatocoenosis”) for this study. A recent morpho- litho-stratrigraphic study carried out on this geological context shows that this formation was formed during the 5c isotopic sub-stage; OSL dating of Unit C-3 gives an age of 100 ± 8 ka [32, 46]. Forty-four Nassariinea shells were collected from a small outcrop in this formation in 2018, and compared with the archaeological samples for US 8.

## Methods

The study of these shells was undertaken during a field campaign by INSAP (*Institut National des Science et de l’Archéologie*, Rabat, Morocco) in 2015. All necessary permits were obtained, within the framework of the scientific partnership agreement between INSAP and MNHN, for the described study, which complied with all relevant regulations.

Taxonomic identifications were undertaken according to previous works [16,26,47,48], reference books [49–54], and internet open access databases (“Données d'Observations pour la Reconnaissance et l'Identification de la faune et la flore Subaquatiques – DORIS” http://doris.ffessm.fr/; “World Register of Marine Species – WORMS” http://www.marinespecies.org/).

The length, width and thickness were measured using a digital caliper for each whole shell and for fragmented ones when possible. Their dimensions were compared with previously published data [15,16]. Statistical tests were performed using PAST Software [55].

**Fig 5:**
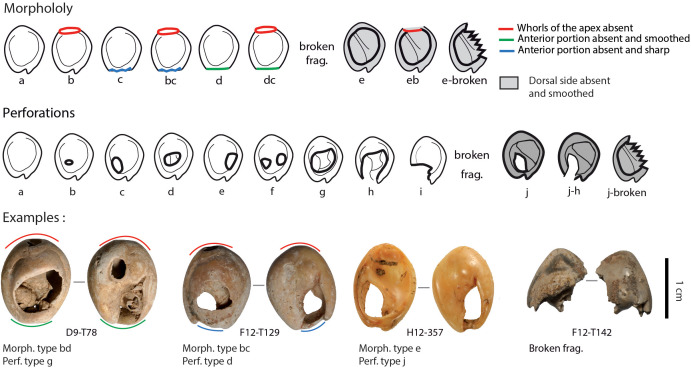
Morphology and perforation types observed for *Tritia* cf*. gibbosula* from US 8 of El Mnasra cave.

**Fig 6:**
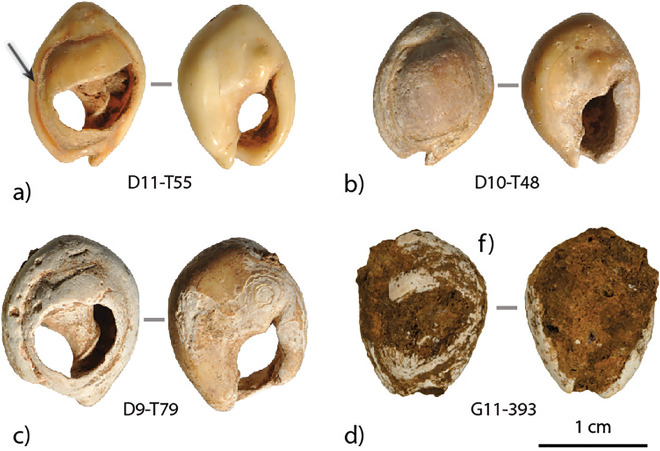
Examples of surface preservation observed for *Tritia* cf*. gibbosula* from US 8 of El Mnasra: a) shiny with natural coloration preserved (see arrow), b) matte, c) chalky, d) chalky encrusted with cave sediment.

For each specimen, the dorsal and ventral sides were photographed (e.g., Figs 5; 6; S1 to S5) and microscopic observations were completed using binocular microscope (x10 – x 80) and low-resolution pictures were made in the field using a Dino-Lite digital microscope. Thirty-one specimens were also exported in order to be investigated at higher resolution using a Hirox numeric microscope (RH-2000) at the Musée de l’Homme, Paris (Plateau d’imagerie 2D/3D du MNHN).

In order to document the evidence of modifications linked to the use of shells as bead ornaments, several features were recorded for each specimen. The presence of fractures and holes on dorsal and ventral sides were recorded according to the classification of perforations based on Vanhaeren et al. [14], Bouzouggar et al., [15] and d’Errico et al. [16], taking into account the previous study published by El Hajraoui et al. [21]. As the sample set is larger than previously published, a wider variability of forms, fractures and perforation types were observed. The categories established in previous studies were thus modified in order to be applied to the entire El Mnasra sample set. More precisely, it was necessary to distinguish modification of the morphology of *Nassarius* shells according to the missing parts (apex, anterior portion, dorsal side), and the location, number and extent of perforations (Fig 5). The morphology of shell beads was classified by 10 categories and perforation type by 13 categories, illustrated in Fig 5. These parameters allowed the recording of all specimens from El Mnasra, but, unfortunately, limited an easy comparison with previous studies. However, a table displaying the correspondence between previous and adapted categories is provided in S2 Table.

**Fig 7:**
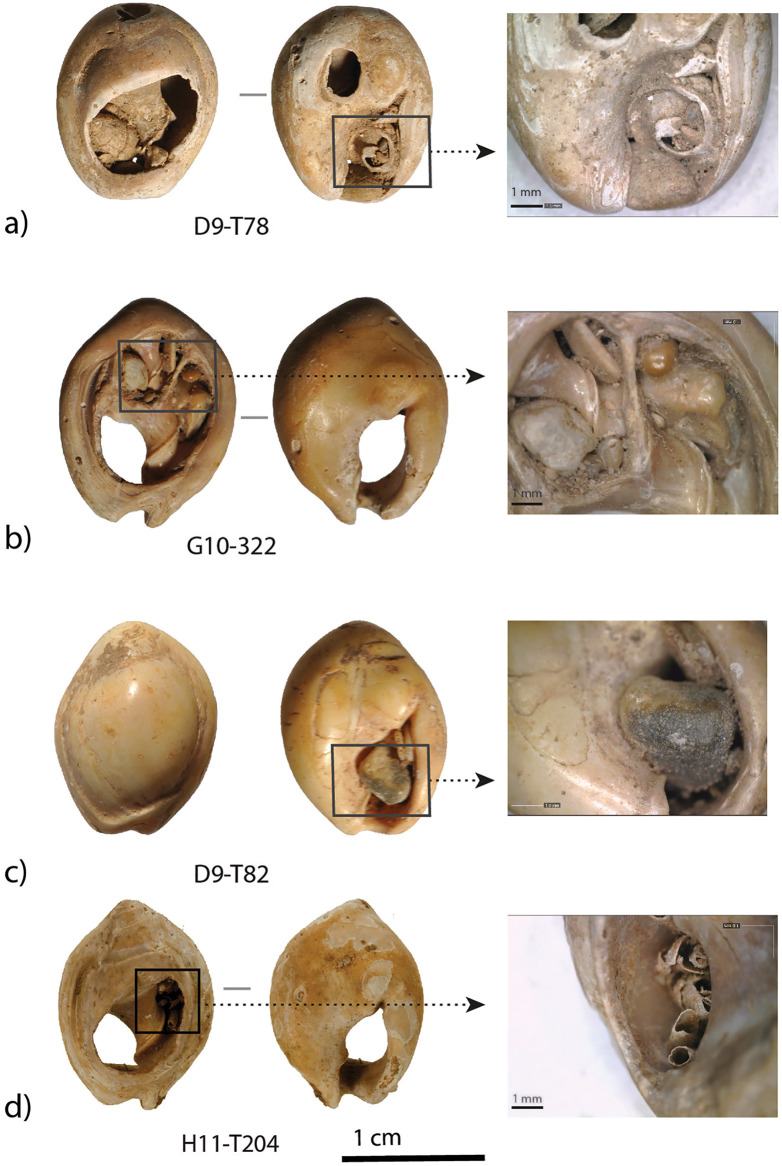
Examples of shell fragments and gravels observed in *Tritia* cf*. gibbosula* shells from US 8 of El Mnasra cave: a) shell fragments and gravels, b,c) gravels, d) Serpulid tubeworm.

Taphonomic features were also documented by recording surface preservation (chalky, matt or shiny; e.g., Fig 6) and encrustations (sediments, gravels or shell fragments; e.g., Fig 7). The heating of shells was identified according to the surface color (grey/black color) and the presence of cracks, desquamation areas, and glossy black coatings and the presence of red pigment residues plus their location was documented. The localization of use-wear (smoothing or facet) was registered and their intensity was coded from 0 (no smoothing or facet) to 3 (intense smoothing or facet). All the information recorded for the paleontological specimens from Dar es Soltane C3 and for the shell beads from the US 8 of El Mnasra cave are provided in a comprehensive table (S3 Table). In order to confirm that the red deposits observed on shell surface contain hematite mineral (“ochre”) and not another red pigment (i.e.,; cinnabar, red organic pigment) Raman spectroscopy analyses were carried out on three representative samples (D11-505; H11-T178; G10-298) using a Labram HR800 spectrometer (Horiba Jobin Yvon) with a 458 nm excitation at MONARIS lab (UMR 8233; Sorbonne Université – CNRS).

## Results

### Taxonomic identification and shell morphology

#### Taxonomic identification.

The majority of the shells beads from the US 8 of El Mnasra present features similar to *Tritia gibbosula* (N = 272; formerly named *Nassarius gibbosulus*; Systematic revision following Aissaoui, Galindo [56]) and/or *Nassarius circumcinctus).* These two species, currently present in the Mediterranean area [57], have been identified in several Aterian archaeological contexts in North Africa [14–17,19–22] and can be differentiated according to their size, morphology and color. However, differentiation of species is difficult for Nassariidae due to weak and apparently continuous variations of shell characters [56]. In addition, the morphology of *Tritia gibbosula* could have changed overtime, meaning that Pleistocene specimens are larger with a thicker parietal shield [15].

Moreover, fossil specimens have been submitted to taphonomic processes, which makes them difficult to differentiate and means the coloration could have vanished or been modified. One of the most discriminating characters between these two species is the apex and the protoconch. Unfortunately, this part is often absent on archaeological specimens; indeed, Bar-Yosef Mayer [26] suggests that *Tritia gibbosula* and *Nassarius circumcintus* may be confused in an archaeological context.

Due to the absence of identification at species level on taphonomically altered specimens, and the actual distribution of the *Tritia gibbosula* and *Nassarius circumcintus,* this study prefers to attribute this set of shell specimens to *Tritia* cf*. gibbosula.*

These species are not currently abundant around Rabat-Témara. However, Chakroun et al. [47] identified *Tritia gibbosula* and *Nassarius circumcinctus* in the natural fossil thanatocenoses at Quarry 10 and Guyville outcrops (Témara-Rabat region, near Kebibat site; see Fig 1b) attributed to Pleistocene MIS 5.5. The natural fossil thanatocenosis at Quarry 10 is particularly rich in Nassariidae. The Nassariidae collected in the present study (Dar es Soltane – Unit C-3 thanatocoenosis”, DeS-C3) do not show a preserved protoconch and did not allow distinguishing between the two species.

In the archaeological material of El Mnasra, six shells displaying several whorls, a columella fold on the aperture, and an outer lip with teeth were identified as another Nassariidae: *Tritia corniculum* (Fig 3). Several specimens of this species were also identified at Contrebandiers Cave, located near El Mnasra [22].

Three shells belonged to Columbellidae, *Columbella rustica* (Fig 3)*.* This is an epifaunal gastropod species herbivore grazer, living on rocky shores, at a infra-littoral level, in shallow water (3‒12 m) [47].

Three shells were identified as Muricidae, *Ocenebra* sp. (cf. *erinaceus*; (Fig 3)).

One *Ocenebra erinaceus* specimen was identified in MIS 5.5 to 5.3 at the Quarry 10 (Guyville) [47]; it is currently present in the Témara region (Campmas, personal observations).

The abundance of these different species in US 8 is summarized in.

For the spatial distribution, no concentration of the different species has been identified, except five *Tritia* cf*. gibbosula* shells found close to one another (Fig 4).

#### Dimensions.

**Fig 8:**
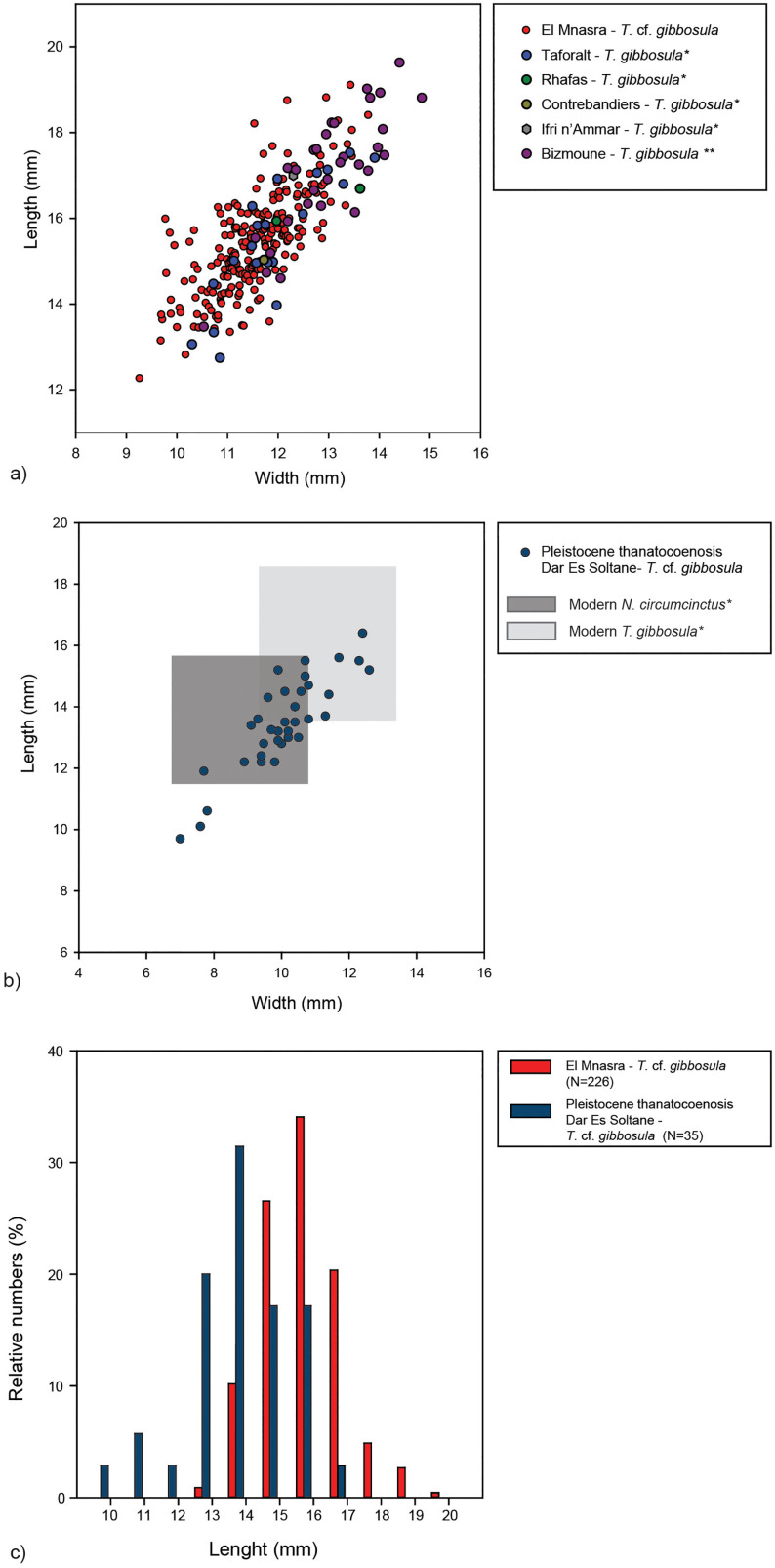
Size of*Tritia* cf*. gibbosula* of El Mnasra cave. a) Size of *Tritia* cf*. gibbosula* identified in US 8 of El Mnasra cave compared to other MSA sites from North Africa (* data from [16], ** data from [12]), b) Size of *Tritia* cf*. gibbosula* from Pleistocene in *Dar es Soltane Unit 3* thanatocoenosis *(DeS-C3).* c) Comparison of size distribution for *Tritia* cf*. gibbosula* from US 8 with those from DeS-C3 thanatocoenosis.

The size distribution of shell beads (N = 284) from US 8 is presented in Fig 8, in comparison with specimens previously published from the Aterian archaeological contexts of Taforalt, Ifri n’Amar, Rhafas and Contrebandiers [16]. *Tritia* cf*. gibbosula* specimens from El Mnasra present a significant size variability (length and width could be measured for 209 specimens of the 272 *Tritia* cf*. gibbosula*).This variability overlaps those observed for specimens from other Aterian contexts (Bizmoune, Taforalt, Rhafas and Ifri n’Amar; [12,15]). There is no significant difference in size between the Taforalt and El Mnasra specimens (t test; p = 0.94), but the Bizmoune specimens are significantly larger (t test; p < 0.001).

The majority of *Tritia* cf. *gibbosula* from the US 8 of El Mnasra are similar in size to *Tritia gibbosula* from the modern specimens of Tunisia and Israel (see Fig 8b - data on modern specimens from modern biocoenosis and thanatocoenosis from [16]), and clearly larger than modern *Nassarius circumcictus.* These observations are consistent with the attribution of Nassarius shell beads from El Mnasra to *Tritia* cf*. gibbosula* rather than *Nassarius circumcictus*.

Both length and width of *Tritia* cf*. gibbosula* form US 8 are slightly larger (t test; p < 0.001) than specimen from the Pleistocene thanatocoenosis (DeS-C3). The size distribution of *Tritia* cf*. gibbosula* coming from DeS-C3 covers the size variability observed for both *N. circumcictus* and *Tritia gibbosula* modern populations (Fig 8b). Pleistocene specimens of *Tritia gibbosula*, specifically from the last interglacial, are generally wider than modern ones [14,16]. This is particularly the case for Tunisian populations from a fossil beach dated to 126 ± 7 ka [16], but is not the case for a large part of the specimens from the DeS-C3 fossil thanatocoenosis dated to 100 ka. It could, however, reflect a misattribution of the species from DeS-C3 (the presence of both *N. circumcictus* and *Tritia gibbosula* specimens), a size variation between Atlantic and Mediterranean populations, or a variation in Pleistocene population size during the last interglacial due to climatic variations.

#### Morphology and taphonomic features.

Morphology: For *Tritia* cf*. gibbosula*, 76.8% of the shells have their global morphology preserved (type a, N = 209; Fig 5; Table 2). However, all shells present a muted sculpture with a smoothing of the tallest spires of the apex. This feature is typical of mechanical wave abrasion on the shore. This smoothing is particularly pronounced for some samples since the apex is absent for 15.8% of the shells (types b, bc, dc, and eb; N = 43) and the anterior portion is absent for 9.6% (types c, bc, d, and dc; N = 26). The apex and the anterior part are absent for 5.5% (types bc and dc; N = 15), resulting in a very rounded morphology. Eight specimens are “flat” (2.3%), meaning that the dorsal side is totally absent (types e-eb-and e-broken). Among these shells, three have no apex (type eb). In addition, four specimens (1.5%) are represented by small fragments with morphological characteristics that allow them to be attributed to *Tritia* cf*. gibbosula*. Smaller fragments could still be present within the US 8 sieve refuse ‒ their identification was difficult due to the large amount of mussel shell fragments.

**Table 2:** Morphological types of *Tritia* cf*. gibbosula* from US 8 (N = 272) of El Mnasra cave compared with the Djerba biocenosis, Dar es Soltane thanatocoenosis, and Taforalt specimens (Taforalt and Djerba data from [15,16]). [a] morphology is globally preserved, [b] apex whorls are absent, [c] anterior portion is absent (broken), [d] anterior portion is absent (smoothed), [e] dorsal portion is absent (“flat”).

		a	b	c	bc	d	dc	Broken Frag	e	eb	e-broken	Total
US8-El Mnasra	N	209	25	7	2	4	13	4	4	3	1	272
	%	76,8	9,2	2,6	0,7	1,5	4,8	1,5	1,5	1,1	0,4	100
Taforalt	N	19	8					5				32
	%	59,4	25,0					15,6				100
Djerba	N	213	8					64				285
	%	74,7	2,8					22,5				100
C3 Dar es Soltane	N	24	9	1	2	1		7				44
	%	54,5	20,5	2,3	4,5	2,3		15,9				100

The natural thanatocenosis of DeS-C3 was characterized by a predominance of shells displaying a preserved morphology (type a; N = 24, 54.5%), the loss of the apex (type b; N = 9, 21.4%), or fragmentation (broken frag.; N = 15, 54.5%).

#### Taphonomic conservation.

Among *Tritia* cf*. gibbosula*, 42.6% (N = 116) of the shells bear brown/orange traces due the encrustation of cave sediments, which adheres to the archaeological material and can limit surface observations (Fig 6). A large number of shells displayed a modern shiny-like aspect (N = 90; 33.1%), though the majority were matte (N = 101; 37.0%) or chalky (N = 81; 29.8%). This final aspect (Fig 6c) is probably linked to post-depositional processes in the cave sedimentary deposits. Almost all the shells (93.7%) have small white pits, which have accumulated into a chalky aspect that can extend over the entire surface. Among the shiny shells, some have natural coloration preserved (Fig 6a). No evidence of damage by predators has been identified.

For other species, four *Tritia corniculum* shells are shiny, one is chalky and one is matte (Table 3). One *Columbella rustica* shell is shiny and two others have at least one chalky portion. The three *Ocenebra* sp. *s*hells are matte.

**Table 3:** Number of *Tritia* cf*. gibbosula* shells from US 8 of El Mnasra cave according to their surface preservation.

Surface preservation	Number	% Number
Chalky	81	29.8
Matte	101	37.1
Shiny	90	33.1
TOTAL	272	

The presence of shell fragments and rounded beach gravels inside the shell was identified for 25 *Tritia* cf*. gibbosula* (9.2%) and 2 *Columbella rustica* shells (e.g., on Fig 7). For *Tritia* cf*. gibbosula*, these internal shell elements have variable morphology and sizes, and are present both in unperforated (N = 9), perforated (N = 14), flat (N = 1) and broken (N = 1) shells. The presence of beach gravels trapped in shells was also identified in the Pleistocene thanatocenoses of Quarry 10 and Guyville [47] and at Taforalt [15].

Shell fragments and rounded beach gravels inside shells confirm their gathering on shoreline after the death of the animal. Among the fragments identified, one specimen seems to belong to a Serpulid tubeworm (Fig 7a). Serpulidae live under the water, indicating that this shell was left submerged for a long time after death, or at least a sufficiently long enough period to allow the development of the tubeworm.

#### Modifications and use-wear.

**Perforations:** Only 13 *Tritia* cf*. gibbosula* shells (4.8%) bear ventral perforations (Table 4). Concerning dorsal perforations, 61 *Tritia* cf*. gibbosula* shells (22.4%) are unperforated (type a), 9 (3.3%) present a small perforation (type b), 2 have a small perforation on the left (0.7%; type c), 49 have central perforations (18.0%; type d), 2 have a small perforation to the right (0.7%; type e), 2 present two perforations (0.7%; type f), 107 (39.3%) evidence a large central perforation (type g), 23 have a large open perforation (8.5%; type h), 5 are broken on the anterior left portion (1.8%; type i), and 4 are broken (for one of them, a perforation is identifiable).

**Table 4:** Perforated shells of *Tritia* cf*. gibbosula (N = 272)* from US 8 of El Mnasra cave compared with the Djerba biocenosis, Dar es Soltane thanatocoenosis, and Taforalt specimens (Taforalt and Djerba data from [15,16]). [a] not perforated, [b] small perforation(s) (if small perforation was associated with another perforation type, it is not indicated), [c] left perforation, [d] central perforation, [e] right perforation, [f] 2 perforations, [g] large perforation, [h] – “open” perforation, [i] broken on the proximal portion, [j] without the dorsal portion “flat”; (Infography E. Campmas).

		a	b	c	d	e	f	g	h	i	Broken Frag	j	j-h	j-broken	Total
US8-El Mnasra	N	61	9	2	49	2	2	107	23	5	4	6	1	1	272
	%	22,4	3,3	0,7	18,0	0,7	0,7	39,3	8,5	1,8	1,5	2,2	0,4	0,4	100
Taforalt	N	3			18			6			5				32
	%	9,4			56,3			18,8			15,6				100
Djerba	N	160	10	8	10	4	4	13		12	64				285
	%	56,1	3,5	2,8	3,5	1,4	1,4	4,6		4,2	22,5				100
C3 Dar es Soltane	N	12			1	4	5	2		11	7	2			44
	%	27,3			2,3	9,1	11,4	4,5		25,0	15,9	4,5			100

Regarding height flat specimens (2.9%) for which the dorsal side is absent, one presents an open perforation similar to type h (type j + h) and one is broken (type j-broken).

The perforation types of the specimens collected in the thanatocoenosis of Dar es Soltane are characterized by an important proportion, i.e., 52.3% of unperforated specimens: whole specimens (type a; N = 12; 27.3%), unperforated and broken specimens (type i; N = 11; 25.0%). Other are fragments (N = 7; 15.9%). Perforations were observed for 12 shells (27.3%; types d, e, f, g). Specimens with a total destruction of the dorsal side were observed in the proportion of 4.5% (N = 2; Type j).

Table 2 summarizes the morphology features of *Tritia* cf*. gibbosula* from the US 8 of El Mnasra (N = 272), in comparison with those from Taforalt, the Djerba biocenosis [15,16], and from the DeS-C3 thanatocenosis. One can note the good preservation of El Mnasra specimens, since the global morphology (type a) is preserved in similar proportions to the Dar es Soltane thanatocoenosis and in significantly higher proportion than in Djerba biocenosis (chi-2; p < 0.001).

Regarding dorsal perforation features (Table 4), the shell beads from El Mnasra are distinguished from those of Taforalt by a wider diversity of perforation types: almost all perforations types observed in the Djerba biocenosis were recognized in the US 8 of El Mnasra. Moreover, the proportions of perforation types for El Mnasra shell beads are significantly different compared to the Taforalt specimens (chi-2; p < 0.001), and the El Mnasra shell beads are characterized by high proportions of large perforations (type g and h) and unperforated shells (type a), and a low proportion of fragments. The latter are most probably due to the difficulty in distinguishing shell bead fragments from the shell fragments from other species in large quantities in the US 8 sieved fraction.

For *Tritia corniculum* (N = 6), five specimens have their morphology broadly intact (type a) and one has the apex absent (type b). For *Columbella rustica* (N = 3), one specimen is intact (type a) and two have the apex absent (type b). *Ocenebra* sp. (N = 3) are intact.

Regarding perforations, all *Tritia corniculum* shells (N = 6) present a large dorsal perforation. For *Columbella rustica,* two present a perforation and one is unperforated. *Ocenebra* sp. shells are unperforated.

*Tritia* cf. *gibbosula* shells from US 8 of El Mnasra therefore show marked differences compared to the Pleistocene thanatocoenosis (DeS-C3), notably larger sizes and the presence of large perforations in significant proportions. These naturally perforated, large shells could have been selected on the beach for use. A study of use-wear is necessary to validate this hypothesis.

**Fig 9:**
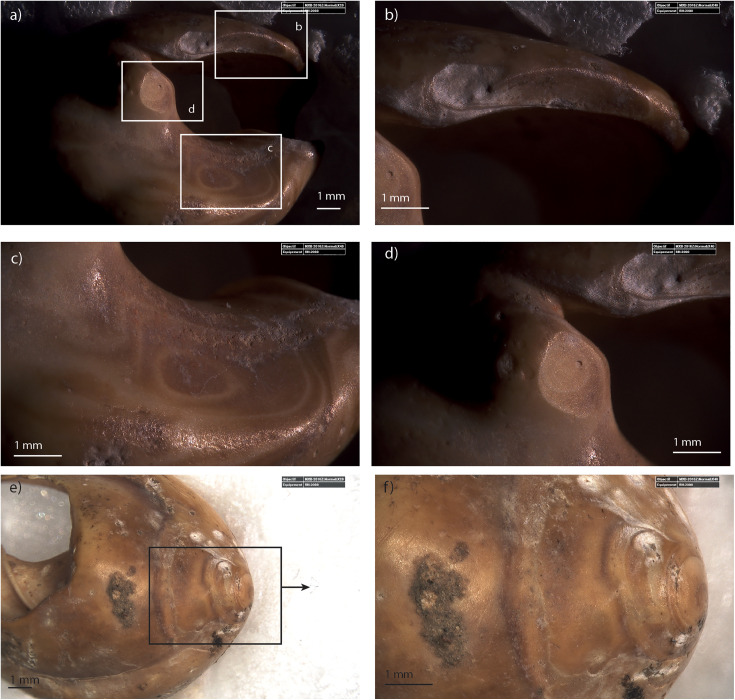
Example of use-wear on *Tritia* cf*. gibbosula* (specimen G11-548) from US 8 of El Mnasra a) general view of the ventral side and details of use-wear facets on the lip (b), on the parietal wall near the aperture and (c), on the hump near the posterior canal (d). A abrasion facet can also be observed on the dorsal side, near the apex (e; f).

**Fig 10:**
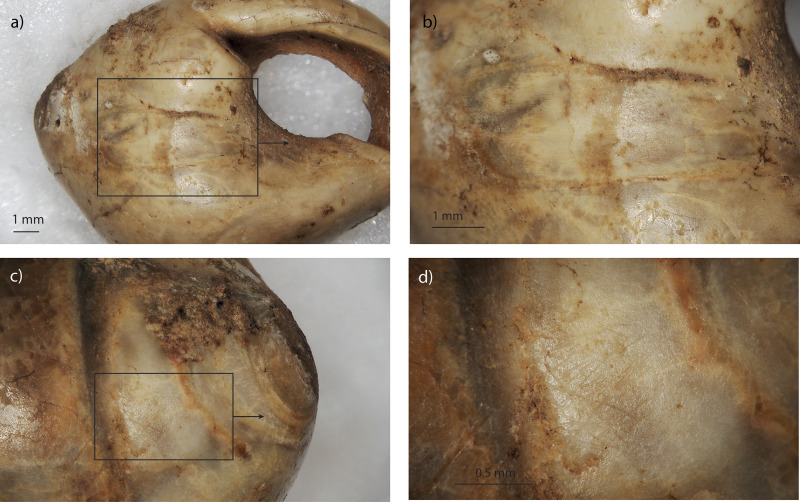
General view of the ventral side for specimen D12-T167 (a), and details of use-wear facets on the ventral side (b) and on the apex (c-d). Abrasion facet on the apex displays a palimpsest of oriented striations (i).

**Fig 11:**
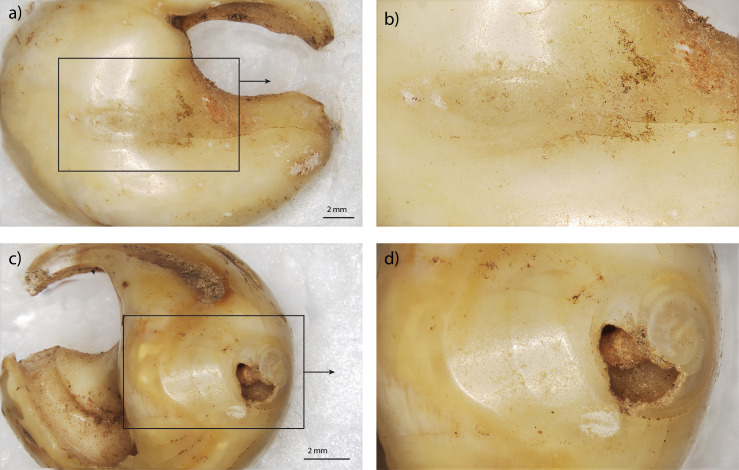
General view of the ventral side for specimen E12-530 (a), and details of use-wear facets on the ventral side (b) and on the apex (c-d).

**Fig 12:**
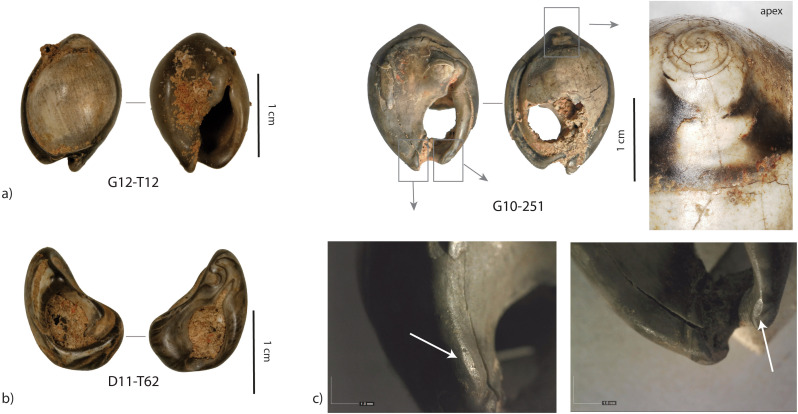
Examples of a) unperforated and b) smoothed fragment of heated *Tritia* cf*. gibbosula* from US 8 of El Mnasra cave. c) Example of heated shell displaying use-wear. On the apex the use-wear overlapping heating features suggests that heated shells were used as beads.

***Use-wear:*** Numerous perforated *Tritia* cf*. gibbosula* presented smoothed perforation edges (N = 130). However, though this feature is difficult to distinguish from wave abrasions, specimens from the DeS-C3 thanatocoenosis do not exhibit features usually attributed to use-wear [1,26,58]. Marine erosion has a gradual smoothing effect on shells, with convex parts being attacked first. This alteration is more or less uniform on perforation edges, combining with smoothing or even breakage of the apex and tallest spire (morphological types b, bc, dc, eb; Table 2). In US 8 of El Mnasra, 46 *Tritia* cf*. gibbosula* are found to exhibit a pronounced smoothing pattern of the perforation edges (level 2 and 3 of smoothing; S1 Table). A less pronounced smoothing pattern of the perforation edge (level 1 of perforation edge smoothing) is observed for 88 *Tritia* cf*. gibbosula* shells and is more difficult to discriminate from wave abrasion. However, for 20 of them, their use as ornaments is corroborated by the addition of very localized use-wear that alters both the edges of the perforation and other areas of the specimen. These abrasions facets are not observed on modern or fossil thanatocoenoses, and their localizations and features are similar to those observed on other archaeological MSA assemblages and experimental samples [1,15,16,59]. These abrasion facets affect 17.3% (N = 47) of the *Tritia* cf*. gibbosula* shells in US 8. They were mainly identified on the ventral side: on the outer lip (N = 39; Fig 9a,b), on the parietal area near the aperture (N = 9; Fig 9c), on a hump near the posterior canal (N = 6; Fig 9d), and in the central part of the ventral side (N = 11;Figs 10a,b; 11a.b), on the columella (N = 4; Fig 5 H12-357). Abrasion facets have also been observed on the dorsal part of the *Tritia* cf*. gibbosula,* near the apex (N = 16; Figs 9e, f; 10c,d; 11c,d). These abrasion facets near the apex are well delimited and present shiny surfaces displaying a palimpsest of randomly oriented striations (Figs 10d; 11d) similar to those observed at Taforalt, and attributed to the scratching of these areas with particles of homogeneous size in a configuration in which the shells are relatively free to move [16]. For one heated specimen, the abrasion facet near the apex erases heating feature (Fig 12c).

These abrasion facets can be clearly distinguished from the natural smoothing induced by wave abrasion which affects all sides of the apex (dorsal, lateral and ventral sides). Moreover, the abrasion facets affecting the central area of the ventral side (Fig 9g,h, S6 Fig) occur in a concave part of the shell and cannot be explained by natural damages which mainly affects raised surfaces.

**Fig 13:**
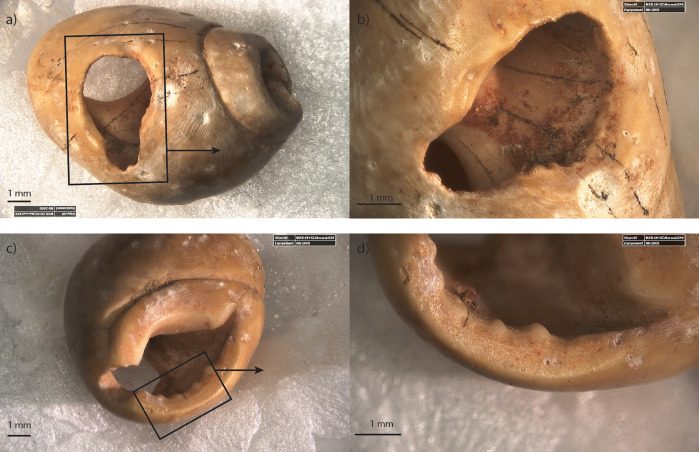
Example of use-wear on a *Tritia corniculum* specimen from US 8 of El Mnasra cave (G12‒T175). (a) General view of the dorsal side, and smoothing on perforation edges (b). (c) View of the aperture and details of use-wear facet on the lip (d).

*Tritia* cf. *gibbosula* is not the only species displaying use-wear: four of the six *Tritia corniculum* perforated shells have use-wear around the aperture (e.g., G12-T175; Fig 13) and one of the three *Columbella rustica* present smoothed perforation edges. Use-wear is probably underestimated because stigmas that were not sufficiently clear and distinctive were not counted.

**Fig 14:**
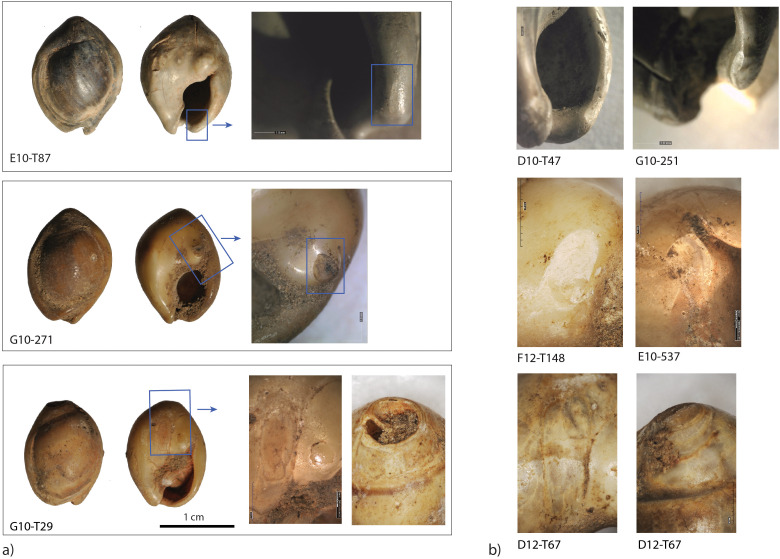
Example of use-wear on unperforated Tritia cf. gibbosula. a) Unperforated *Tritia* cf. *gibbosula* from the US 8 displaying abrasion facets b) similar abrasion facets observed on perforated specimens in the US 8 of El Mnasra cave.

**Fig 15:**
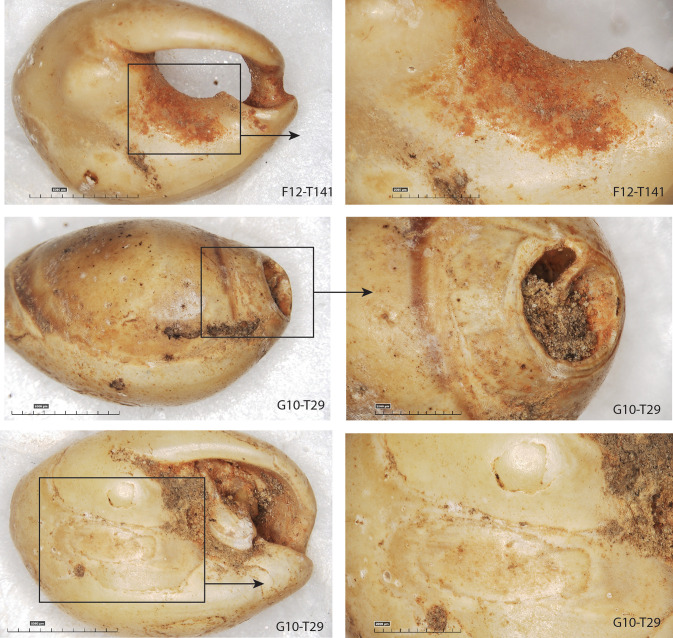
Examples of pigment residues on *Tritia* cf*. gibbosula* shell from US 8 of El Mnasra cave. One unperforated specimen (G10-T29) presents pigments and abrasion facets on its dorsal and ventral sides.

Interestingly, one unperforated specimen of *Tritia* cf*. gibbosula* displays a use-wear facet on the lip (G10-271; Fig 14a) and two others present an abrasion areas on the dorsal side near the apex and on the concave area on the ventral side (E10-T87, G10-T29; Figs 14, 15). These use-wear patterns are similar to those observed for perforated specimens.

*Heating modifications*: Experiments conducted by d'Errico et al. [60] show that shells artificially heated in a reductive environment rich in organic material displayed a homogeneous black color whereas heating in an oxidizing atmosphere produced more variable colors changing from beige (200°C), brown (300°C), black (400°C), gray (500°C and white (800°C). Among the *Tritia* cf*. gibbosula* shells from US 8, 45 specimens (16.5%) present dark brown to gray-white colors and heating cracks (Fig 12). Several unperforated shells (N = 14) and the one totally smoothed fragment had also been heated (Fig 12 a and b). Even if the beige/brown coloration may not have been identified due to a color too similar to the natural one, the presence of brown, black and light gray shells in US 8 suggest heating in oxidizing condition. This is different from what has been suggested for shell beads with a homogeneous black color at Blombos and Taforalt, which have been interpreted as having been deliberately heated in a reductive environment rich in organic material [16,60]. At El Mnasra, it is questionable whether the shells were intentionally heated, given the number of fireplaces and burnt faunal remains uncovered in US 8 [29,30,40]. However, several heated shells present use-wear (N = 7; 15.6% of the heated *Tritia* cf*. gibbosula*), and the presence of use-wear overlapping heating features suggest that heated shell were used as beads (e.g., on the apex of specimen G10-251; Fig 12 c). These observations are consistent with those made at Taforalt where use-wear on a heated shell suggest that shells blackened by heating were used as beads [16].

**Fig 16:**
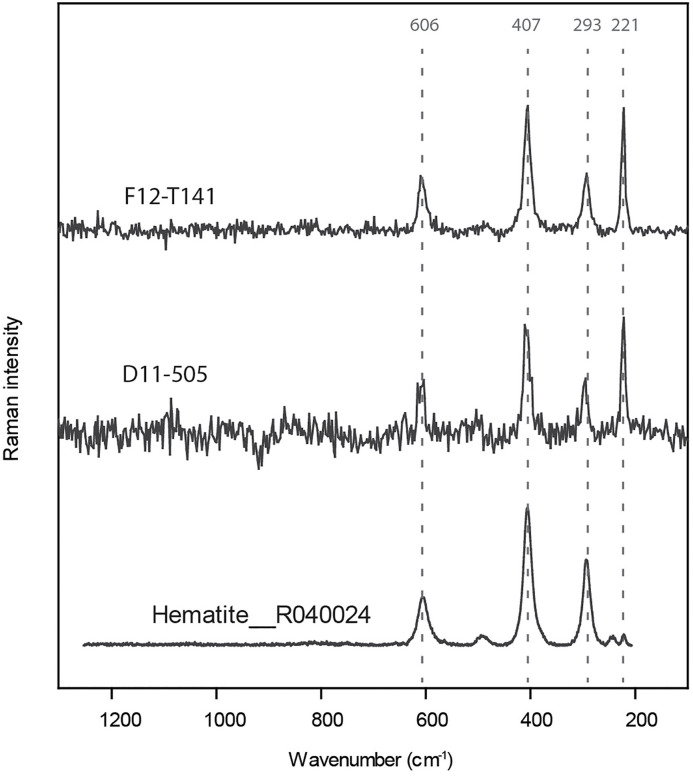
Raman spectra of pigment covering shell beads F12-T141 and D11-505 (*Tritia* cf*. gibbosula*) compared to reference Raman spectra (R040024) of hematite from RRUFF spectral database [61]. The Raman spectra display the typical peaks of hematite around 606, 407, 290 and 221 cm^-1^.

*Pigments:* If macroscopic studies might lead to confusion regarding “ochre” deposits with brown/orange traces due to encrustation by cave sediments, microscopic observation at a higher magnification allows us to discriminate them from red ochre. Ochre residues are characterized by a very fine grain size and intense color (Fig 15). Raman spectroscopy analysis performed on two shell beads (F12-T141 and D11-505) indicated that these red deposits are composed of pure hematite, as shown in Fig 16. Excavations at El Mnasra did not reveal any ochred layers, and the faceted blocks of pure red hematite identified in US 8 [44] are too few in number to induce accidental contact with shell ornaments. Moreover, the majority of lithic artifacts and bones were not affected by ochre deposits, except for few Aterian pedunculate points and bone tools. In US 8, among shell beads bearing red ochre residues, 89% are perforated (70% for shells without ochre), and 28% display use-wear facets (11% for shells without ochre). These proportions deviate from the expected theorical values (Chi2; p < 0.01) and demonstrate a relationship between the presence of pigment, and the presence of both perforations and use-wear facets. Therefore, the presence of these red ochre residues can be interpreted as resulting from a prolonged contact with an ochred support, as it has been proposed for comparable specimens at Taforalt, Rhafas and Ifri n’Ammar [16].

Red ochre residues were identified on the shells of 49 *Tritia* cf*. gibbosula* specimens (18%) and are present on all portions of the shells: between and around the aperture on the ventral side (N = 23 on the outer lip, N = 32 on the ventral side near the inner lip, N = 22 on other parts of the ventral side), inside the shell aperture (N = 16 on the columella), and on the dorsal side (N = 32). However, thick red ochre deposits were only observed on the ventral side, in the concave part surrounding the aperture where they were probably protected from post-depositional alterations (e.g., F12-T141 on Fig 15: G10-T29 on Fig 14). Five unperforated specimens display traces of ochre, and for one of these specimens the red residues are associated with the presence of use-wear (G10-T29; Fig 15). One *Tritia corniculum* perforated shell contains thick residues of ochre on its columella (Fig 13b).

## Discussion

### Origin and selection of shell beads

On the coastline near the El Mnasra Cave, several geological sequences dated to MIS 5.5 and MIS 5.3 reveal the presence of Nassariidae in their malacofauna assemblages [46,47]: Quarry 10 (Rabat), Guyville (Témara), and near the Dar es Soltane caves (Rabat).

As previously mentioned, *Tritia* cf*. gibbosula* specimens from the Dar es Soltane thanatocenosis dated to 100 ± 8 ka show an important size variability, as well as the presence of smaller than previously described specimens from the thanatocoenosis in Tunisia dated to 126 ± 7 ka [16]. This could reflect a misattribution of the species at Dar es Soltane, since *N. circumcictus* are smaller than *Tritia gibbosula* and the two species are difficult to discriminate. It could also result from a size variation between Atlantic and Mediterranean populations, or a variation of Pleistocene population size during the last interglacial due to climatic variations. A study of Pleistocene geological formations that have yielded Nassarridae remains on the Atlantic and Mediterranean coastline of North Africa could allow for a better understanding of the size variations observed between fossil populations, enabling the criterions of the anthropogenic selection during gathering to be identified. Regardless, the absence of small specimens in the archaeological record of El Mnasra suggests that “adult” (or “sub-adults”) and large specimens were preferentially gathered by Aterian populations.

The presence of shell fragment or gravels inside a small proportion (9.2%) of the *Tritia* cf*. gibbosula* specimens demonstrates their being gathered after the death and subsequent exposure to waves, as already observed at Taforalt [15]. However, as the surface is well preserved for a large part of the specimens, and the global morphology (type a) is preserved in similar proportions to the Djerba biocenosis, one could argue for the gathering of specimens from the zone beach straight after their death. The global slight smoothing observed on the entire surface of El Mnasra’s shells is also indicative of such collecting.

As proposed for the Taforalt specimens, the proportions observed for the different perforation types at El Mnasra contrast with those from the biocenosis and thanatocoenosis. The observed proportions cannot be explained by the random selection of seashells on the beach because, in this case, the proportion of unperforated shells would have been greater. However, the shell beads from El Mnasra are characterized by a wider diversity of perforation types than at Taforalt, since almost all the perforations types observed in the Djerba biocenosis were recognized in US 8. Moreover, the proportion of unperforated specimens (type a) is more than two times higher than at Taforalt. These characteristics bring the El Mnasra specimen features closer to those of a natural biocenosis.

The prevalence of perforated specimens observed for the other MSA assemblages is less important at El Mnasra and is clearly related to the proximity of important shell sources available just at a short distance from El Mnasra, contemporaneous with the occupation of US 8 (see for example in “sequence 3” at “Carrière 10”; Fig 6 in [47]). However, it puts into question the intensity of the anthropogenic selection of shells with holes from the shoreline. The larger proportion of perforated specimens on a site some distance from the coast could be related to the remoteness of the primary sources. But it could also be indicative of the modifications and uses made over the potentially “Long Life” of personal ornaments, in close relation to the mobility of groups of people between gathering and archaeological sites.

A previous study on faunal remains shows that the Aterian populations at El Mnasra exploited marine resources, especially mollusks (limpets and mussels; [29]). In the US 8 of El Mnasra, the low density of artifacts, the small number of exploited species, and the fragmented system of reduction have been interpreted as the testimony of brief occupations possibly related to marine resource exploitation [29]. In this context, the abundance of Nassariidae shells in the Témara region could have been an attractive resource for Aterian populations and may explain the richness of shell beads in US 8 deposits.

The taphonomic study suggests that *Tritia* cf*. gibbosula*, *Tritia corniculum*, and *Columbella rustica* were gathered as shells (without the flesh), but that *Ocenebra* sp. were intact. However, the fact that they display no evidence of use means that they were probably gathered when they were still alive, although their small size argues against them being consumed. These mollusks could have been brought back with their catch; for example, with the mussels and limpets that were consumed by the Aterian of US 8 [29,30].

It seems, therefore, that there is no subsistence and symbolic link. Several species were brought back alive to be consumed later (Patellidae, Mytillidae, Trochidae and Muricidae), while several small gastropods (Nassariidae and Collumbidae) had purely an ornamental function. The consumed mollusks and shells used as ornaments belong to a different species gathered in different biotopes: the consumed mollusks lived on rocky shores, while “ornamental shells” were gathered on the beach. However, these areas were probably quite close to one other on the shoreline, such as in the Témara region, where creeks containing beaches form lagoon-like environments that cut into the rocky shore [30]. Nevertheless, although very close, these different zones present a variety of constraints that would have led to a specialization of activities within the groups. Indeed, the preliminary ethnological observations of actual mussel gatherers on the Rabat-Témara (ACoAPass project, Dir. E. Campmas) shoreline show that this activity is highly dangerous, due the abrupt cliffs swept by powerful Atlantic waves, and is therefore carried out exclusively by experienced adults in good health [62].

### Use of shell beads

A previous study, focusing purely on the morphology and location of perforation was published by El Hajraoui, Oudouche [21]. Our results confirm some of the initial observation regarding the presence of several other species and the diversity of perforation types. However, they also provide a more detailed analysis concerning the different types of perforation and the study of use-wear observed on the shell beads.

The morphologies and perforations are similar to those observed for actual thanatocoenoses, with gravels or shell fragments being trapped inside *Tritia* cf*. gibbosula* shell beads. These observations indicate that a large number of shells were collected already dead, and may have already been naturally perforated on the shore before they were collected. However, deliberate perforations could have been carried out, as previously proposed by El Hajraoui et al. [21] at El Mnasra, or by Stiner et al. [63] for the Üçagızlı Cave shells. Unperforated specimens are at least two times more abundant in the US 8 of El Mnasra Cave than in other MSA assemblages, both in North African [12,14–16] and South African archaeological contexts [1,59]. This raises the question as to the usefulness of gathering unperforated specimens. The proximity of natural sources could have favored an opportunistic gathering on the beach, following by the subsequent selection of perforated shells in the campsite and the discarding of unperforated ones (a “raw material high-grading explanation”; Stiner, Kuhn [63]). Another explanation could be that they were gathered for future perforation. In the case of the first examination of this collection, no traces or specific features that could confirm this hypothesis were identified. A more complete analysis of the material would be necessary to determine if any intentional perforation has taken place. A third explanation should not be neglected: the possibility that unperforated shells were selected, transported and used by Aterian populations because they constitute items with symbolic significance, although they were used in a different way than ornaments, or because they were used as ornaments in a design that did not require a perforation, for example fixed on another support. Indeed, several unperforated specimens bear traces of pigments (N = 5; E10-T91, F12-T132, G10-T29, G12-538, G12-T211) and three display facets or localized abrasion area (N = 3; G10-271; E10-T87, G10-T29). These traces are localized, unlike natural marine erosion traces which are more widespread, and are similar to those observed for perforated specimens. These traces of use suggests that a fixing system was employed, such as a bond placed between the apex and the siphonal canal, fixing the shell along its longitudinal axis (see the longitudinal abrasion on the concave ventral side (G10-T29; Figs 14; 15, S6 Fig). Another possibility was the use of a small gravel used to block a bond in the aperture (e.g., D9-T82 Fig 7; 9 of the 61 (14,7%) unperforated specimens in US 8 of El Mnasra; 2 of the 3 unperforated specimens at Taforalt).

For perforated specimens, use-wear consists mainly of the smoothing of the perforation edges and abrasion facets of the apex on the dorsal side, and the parietal wall and outer lip of the ventral side. These types of use-wear have also been identified on perforated *Nassarius kraussianus* from the Blombos Cave and on experimental beadwork [1]. Even if stringing methods need a detailed study of use-wear associated with experimentation, in the experimental and archaeological data provided by Vanhaeren, d' Errico (1) on Blombos shell beads allow us to hypothesize bead arrangements from use-wear patterns observed in US 8. The smoothing of perforation edges on the dorsal side, associated with use-wear facets on the lip and on the parietal wall of the ventral side, are similar to those observed by Vanhaeren, d' Errico [1] for an arrangement of continuous stringing with alternate orientation.

Another type of use-wear pattern that was identified on several specimens corresponds to a polished facet on the dorsal side apex, associated with an abrasion facet in the central part of the ventral side. It could fit with a second-type arrangement using continuous stringing and the same orientation as described by Vanhaeren et al., [1].

These abrasion facets were only observed for a small number of US 8 shell beads (N = 47; 17.3%), whereas a larger proportion presented less clear evidence of use such as smoothed and polished perforation edges (N = 130). Among these perforated shells without abrasion facets, 35 specimens bear residues of ochre. This could be related to other bead arrangements limiting the development of use-wear as smoothing around the dorsal side perforation; for example, floating pairs of dorsally joining shells, or continuous stringing with the same orientation.

**Fig 17:**
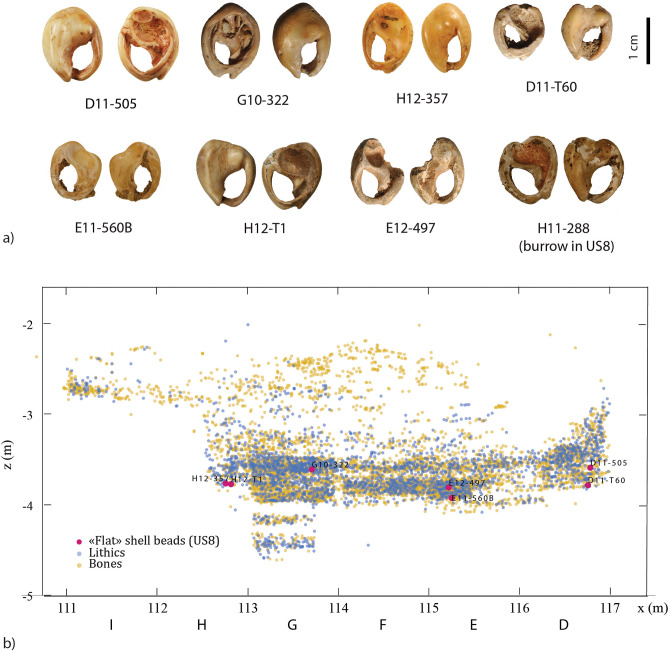
a) Photograph of flat shell beads from US 8 of El Mnasra cave, b) stratigraphical location of flat shell beads (H11-288 from a burrow in US 8 was not plotted).

The US 8 shell beads assemblage is also characterized by the presence of flat specimens (Fig 17a) with a completely abraded dorsal side. This feature is observed in natural assemblages along the Mediterranean coast [63] and in several Upper Palaeolithic sites of this region [64,65]. However, this feature is absent from the MSA archaeological contexts of South Africa [1,59] and the Levant [14–16], but is observed for two specimens at Taforalt (shell beads 9 and 16; Fig 2 in [16]). Such flat shell beads were also described at Timor-Leste archaeological sites dated to 6500 years ago [58]. Here, the technological and use-wear analyses indicated that the dorsal side had been deliberately removed in order to be used as “appliqué,” attached on textiles or other items, as has been observed in ethnographic examples using Nassariidae shells [58]. In our case, it is not possible to determine if their shape is natural or if the dorsal site was deliberated removed, or even if they were used as “appliqué”. However, their smoothed and polished edges, as well as the presence of ochre residues on their surface, argue for intensive use or wearing. The presence of unperforated specimens displaying use-wear facets associated with pigments, for some, reinforces our hypothesis that some *Tritia* cf*. gibbosula* were not only used as beads but were also attached to organic items in an arrangement that doesn't necessarily require perforation.

Several use-wear patterns were recognized in US 8, suggesting that various arrangements and use modes of shell beads were present. At the Blombos Cave, changes of bead arrangement patterns were observed in the MSA sequence [1,66] that may have been due to changes of cultural norms in a community, or the replacement of one community by another. At El Mnasra, flat specimens seem to be localised in the deeper part of US 8 (Fig 17b), especially those with a truncated apex (D11-T60, E11-560B, H12-T1, and E12-497).

However, El Mnasra’s stratigraphic context is different to that of Blombos Cave. At Blombos, a large part of the beads was found in groups of 2–12 specimens. These bead clusters offers the possibility of treating these groups as possible “single beadwork items” [1]. At El Mnasra, although the number of Nassariidae shells is higher, only one group of five beads was found during excavation. Indeed, the thickness of the US 8 is about 50 cm and correspond to a deposit of sandy clay and sand with poorly expressed bedding in which lenses, induced by erosive events (runoff), interlocked. Some subdivisions of this unit may have evolved into structured paleosols due to the actions of meso- and macro-fauna and anthropic action (trampling). This sedimentary context makes it thus difficult to investigate beadwork arrangements from the use-wear patterns, and to raises the question of the continuity or change in bead styles and stringing methods.

As previously mentioned, ochre residues were identified for 18% of *Tritia* cf*. gibbosula* specimens in US 8 and their presence can be linked to their use as ornaments. The presence of ochre residues has been highlighted for previously published MSA shell beads [1,14–16,26,59] and several interpretations were suggested for what is generally attributed to their contact with ochre materials whilst in use, for example as beadwork, with the human body or tanned animal skins coated by ochre [16,67,68].

## Conclusion

At El Mnasra, the shell beads are almost exclusively located in US 8. The recent stratigraphical and chronological model dated the US 8 from 115−94–102−74 ka (95% probability) [24]. The shell beads recovered from the MSA archaeological levels of the Rabat-Témara region in Morocco (El Mnasra, El Harhoura 2 and Contrebandiers caves), therefore, are one of the oldest assemblages, alongside the Bizmoune assemblage [12], highlighting the role of North Africa in the archaeological documentation of first personal ornaments.

The absence of shell beads in the US 9 deposits means that the use of shell beads as personal ornaments appeared after MIS 5.5 at El Mnasra. The small number of shell bead ornaments in USs 7-6-5 (N = 9) indicate the disappearance of shell bead use after MIS 5.1 [24] and might be related to a potential cultural discontinuity between MIS 5 and MIS 4/3 [16]. This suggests that the use of Nassariidea shells as beads might represent a “brief” and ephemeral cultural event, chronologically positioned between MIS 5.5 to MIS 5.1 in this part of Atlantic North Africa.

Including *Tritia* cf*. gibbosula (N = 272), Tritia corniculum (N = 6)* and *Columbella rustica (N = 3)* shells*,* 154 specimens from the US 8 present at least one evidence of use (smoothing of the perforation edge, facet of abrasion, or traces of pigment). Considering the broad meaning of the term ‘ornamental shell,’ which includes not only the specimens used but also the raw material brought to the site by humans with a view to its later use [63]. The shell bead assemblage of the US 8 of El Mnasra cave, with 281 specimens, is the largest when compared to the other main MSA assemblages: Contrebandiers (n = 151), Blombos (N = 68), Taforalt (N = 36), and Bizmoune (N = 33) [1,12,15,16,19,59].

Compared to other North African sites, the shell bead assemblage from El Mnasra presents a number of common features and is thus clearly part of a North African cultural context. However, the El Mnasra shell bead assemblage also presents some specificities. *Tritia* cf*. gibbosula* was the most frequent species at the nearby Témara region sites and Taforalt (the distinction between *Tritia gibbosula* and *Nassarius circumcinctus* not being possible), although *Tritia corniculum and Columbella rustica* species were also used as shell ornaments. The shell beads also displayed similar use-wear as in the other MSA assemblages, but the large number of specimens at El Mnasra made it possible to observe specific use-wear facies, which are infrequent on other sites and which may correspond to a wider range in the modes of ornament use than previously observed. This is especially the case for unperforated and flat specimens that could have been attached (“appliqué”) to other items without having been necessarily perforated.

Finally, the specimens from El Mnasra present similar features (taphonomic alteration, morphology) than at other sites, but the frequency of forms and types of perforations is different to inland sites such as Taforalt. The abundance of perforated specimens observed for the other large MSA assemblages is less significant than at El Mnasra. This is probably related to the proximity of shell sources near El Mnasra, but questions the supposed important selection of naturally perforated shells on the shoreline. In fact, it suggests the notion of the “long life” of shells used as mobile symbolic items, particularly beadwork, which may have been transformed and used several times between the time of gathering and being left on the archaeological site.

The analyses and results presented in this study provide an opportunity to test and refine certain details of the multistep scenario of the human body culturalization proposed by d'Errico *et al.* [10], taking into account the age and specific features of Palaeolithic body ornaments known in North Africa. The characteristics of the Aterian shell beads from US 8 at El Mnasra correspond to a relatively brief event (“ephemeral practices”) at the beginning of the “Third Stage” [10], during which perforation (voluntary or not) and the use of beadwork could have been associated with other modes of use, such as being applied to objects or fixed by a bond without perforation. The El Mnasra shell beads are chronologically situated at the end of the second “major tipping point”, at the beginning of the Upper Pleistocene [10]. In terms of quantity and variety, shell beads from US 8 at El Mnasra document, in an unprecedented and detailed way, the complexity of techniques involved in the production, use and modification of potentially “long life” cultural objects that were operated by mobile human groups in geographically and symbolically structured territories.

## Supporting information

S1 Fig Dorsal (left) and ventral (right) views of Nassariidae specimens from US 8 of El Mnasra cave, in squares D9, D10, D11, and D12. (JPG)

S2 Fig Dorsal (left) and ventral (right) views of Nassariidae specimens from US 8 of El Mnasra cave, in squares E10, E11, and E12. (JPG)

S3 Fig Dorsal (left) and ventral (right) views of Nassariidae specimens from US 8 of El Mnasra cave, in squares F10, F11, and F12. (JPG)

S4 Fig Dorsal (left) and ventral (right) views of Nassariidae specimens from US 8 of El Mnasra cave, in squares G10, G11, and G12. (JPG)

S5 Fig Dorsal (left) and ventral (right) views of Nassariidae specimens from US 8 of El Mnasra cave, in squares H11, and H12. (JPG)

S6 Fig Dorsal view (left), anterior view (middle), and ventral view (right) of two *Tritia* cf*. gibbosula* shells (G10-T29 and D12-T167). Localization of abrasions in a concave part of the ventral side.
(JPG)

S1 Table Descriptive data of shell bead specimens from US 8 of El Mnasra cave. ID, sample identifier; Complete width, height and thickness (if preserved Y (Yes), of uncomplete N (No)); Morphological type – according to Fig 5; Incrustation (sediment; Y if present); Perforation types: according to Fig 5; Smoothing of the perforation edge (from low (1) to high intensity (3)); Use-wear/Facet (Y if present); localization and intensity of use-wear (from low (1) to high intensity (3)); Heating (Y if present); Presence of pigment (Y if present); Pigment localization and intensity from low (1) to high intensity (3).
(XLSX)

S2 Table Descriptive data of *Tritia* cf*. gibbosula* from the Pleistocene thanatocoenosis of Dar es Soltane – Unit C3 (DeS-C3). ID, sample identifier; Morphological type – according to Fig 5; Perforation types: according to Fig 5.
(XLSX)

S3 Table Relationship between Nassariidae shell bead perforation types described by d' Errico, Vanhaeren (16)(Fig 3), and shape and perforation types in the present study. (DOCX)

S4 Table Synthesis of taphonomic and use features for *Tritia* cf*. gibbosula, Tritia corniculum, Columbella rustica and Ocenebra sp*. shells from US 8 of El Mnasra cave. Surface preservation (Shiny, Matte of Chalky), presence of natural alteration: Encrustation by sediment; presence of gravel or shell fragment inside shell; smoothing of the apex, abrasion of the apex (morphological types a, bc, dc, eb), abrasion of the anterior portion (morphological types c,bc,d,dc) by wave abrasion; Smoothing of the perforation edge; Smoothing intensity of the perforation edge (Low (1) to High (3)); Usewear localization; Heating; Presence of ochre; Localization of ochre.
(XLSX)
