# Supplementary figures and images for: Aterian shell beads from the coastal site of El Mnasra Cave (Rabat-Témara, Morocco): Specificities of the north African MSA personal ornaments
Source: PLoS One. 2026 Mar 25;21(3):e0338785. doi: 10.1371/journal.pone.0338785 (PMC13016355; doi:10.1371/journal.pone.0338785)

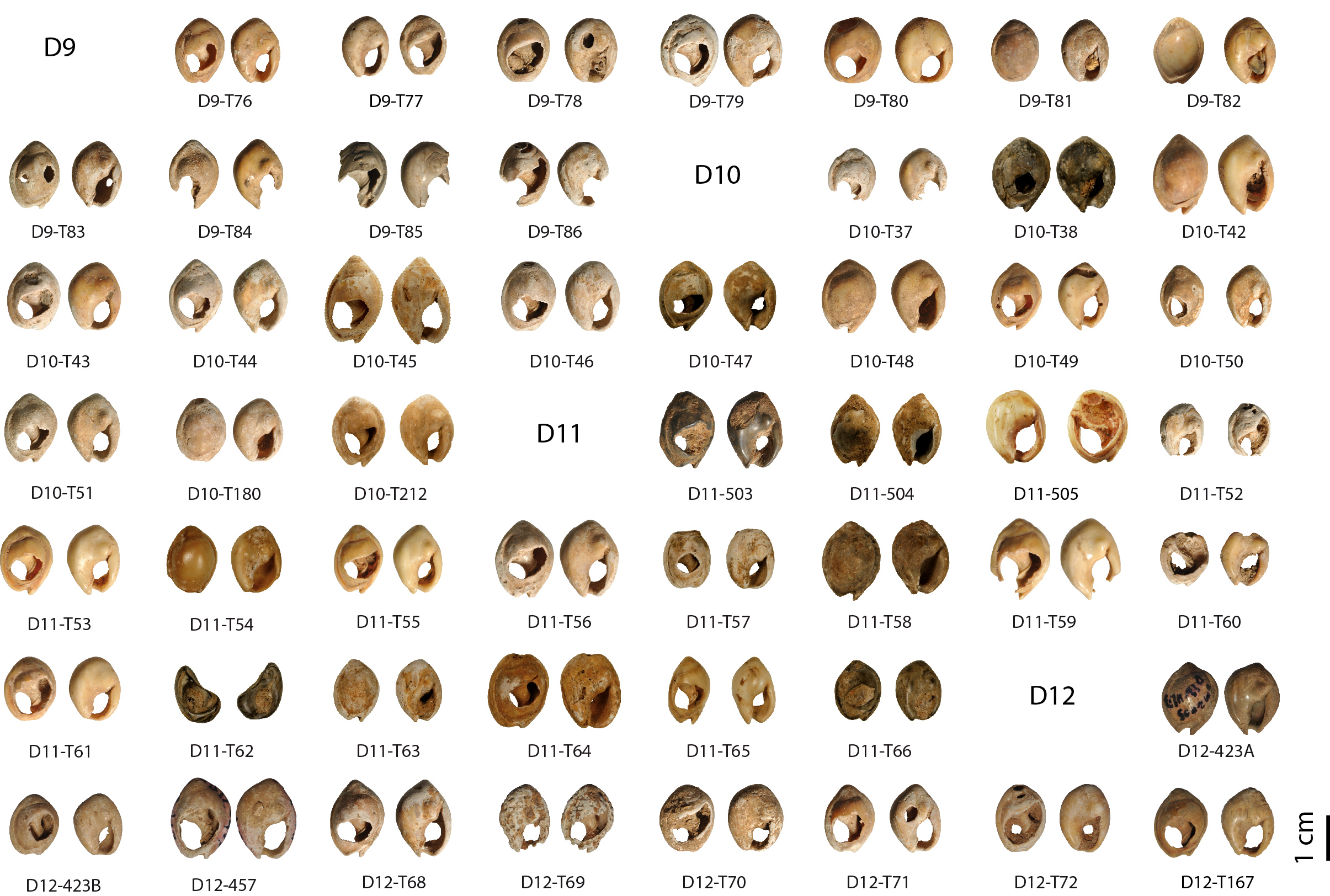

Supplement: S1 Fig — (JPG) [file pone.0338785.s001.jpg]

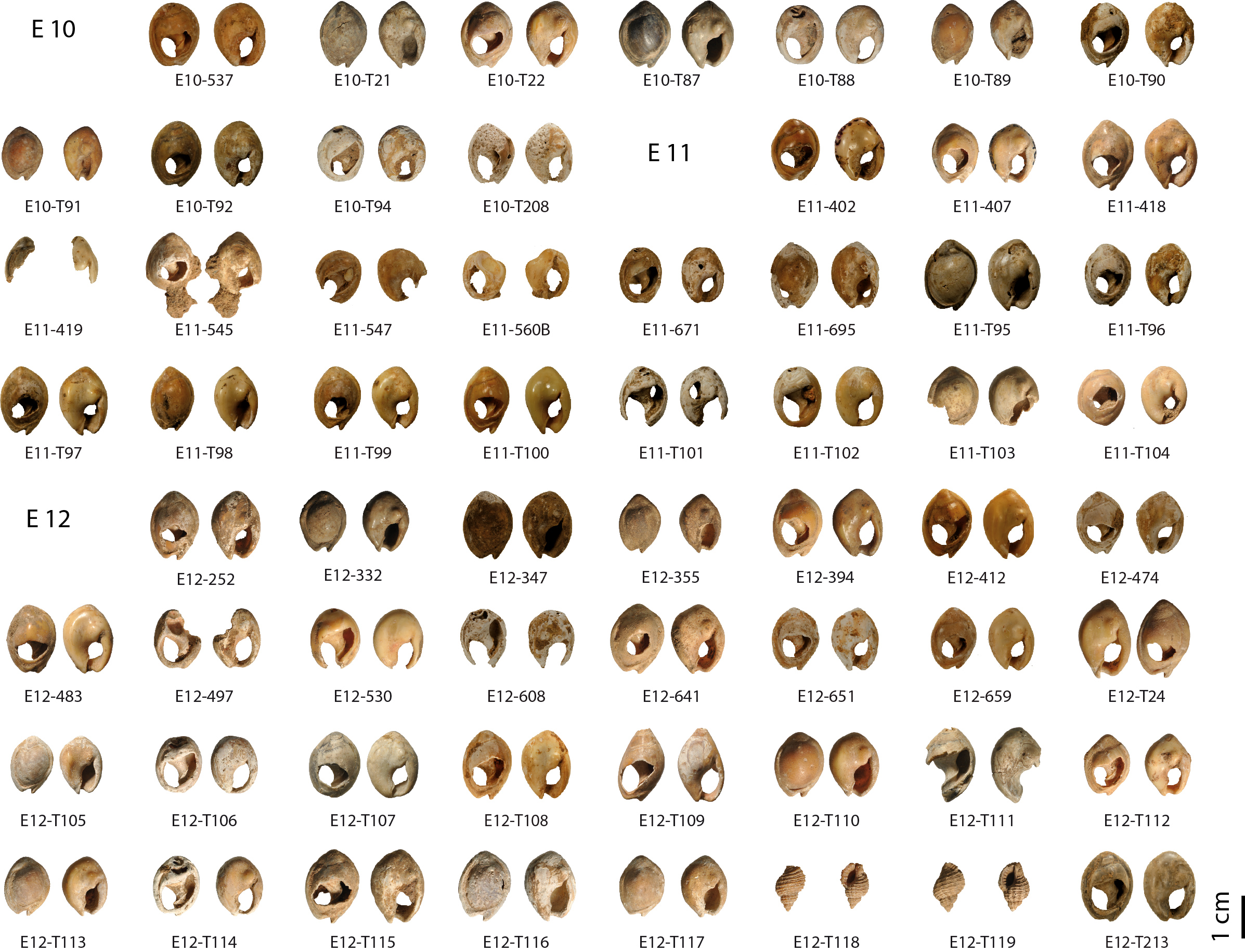

Supplement: S2 Fig — (JPG) [file pone.0338785.s002.jpg]

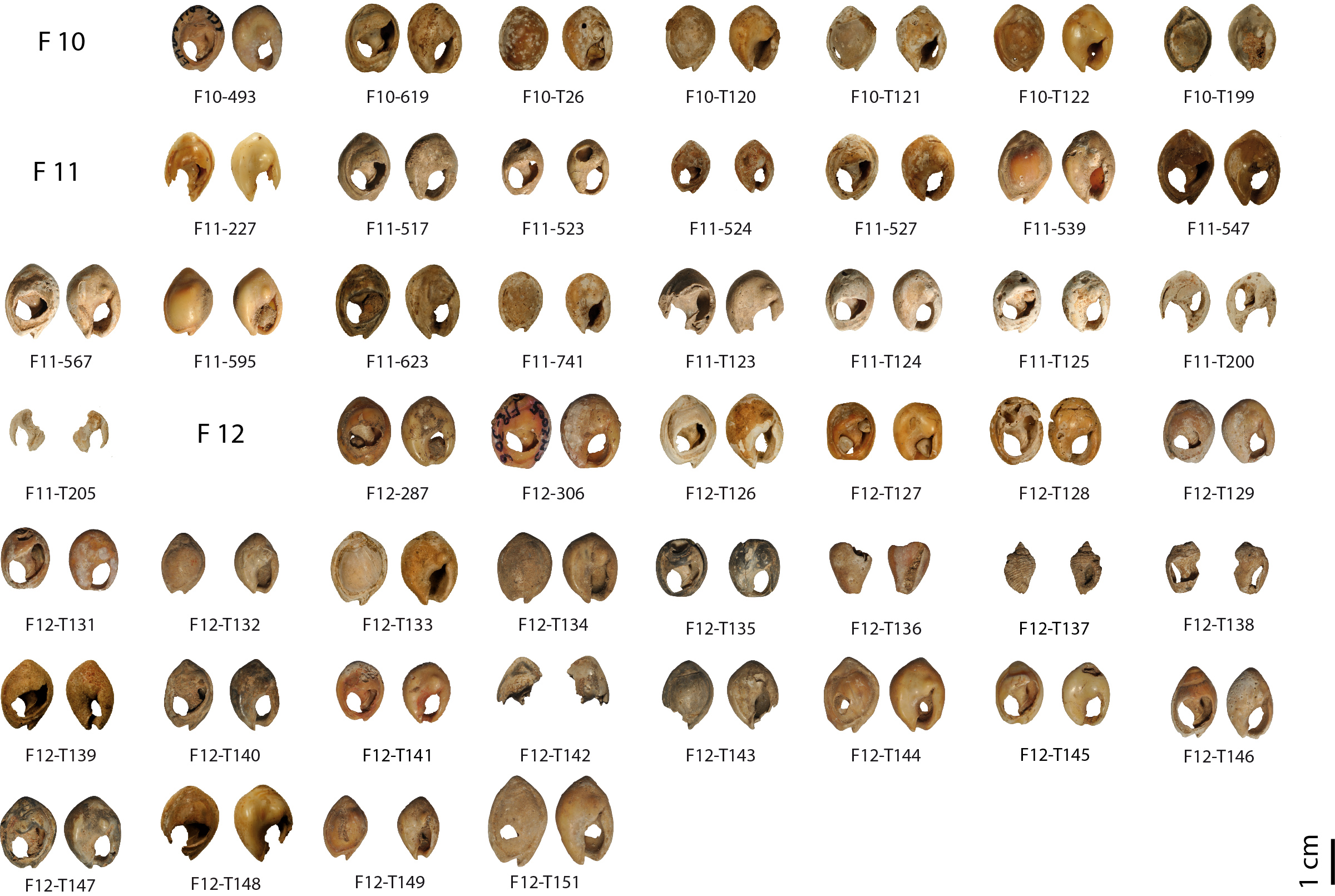

Supplement: S3 Fig — (JPG) [file pone.0338785.s003.jpg]

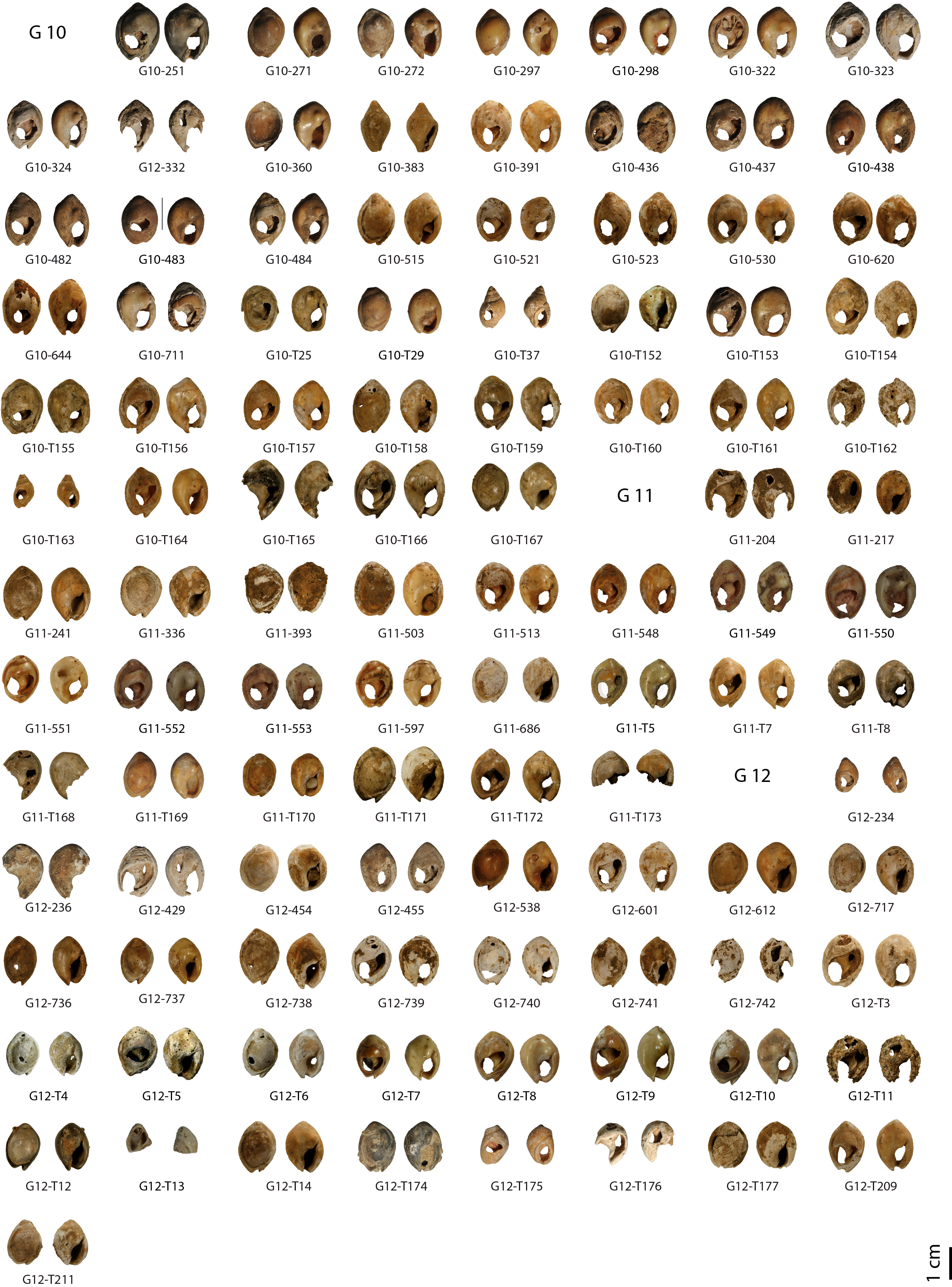

Supplement: S4 Fig — (JPG) [file pone.0338785.s004.jpg]

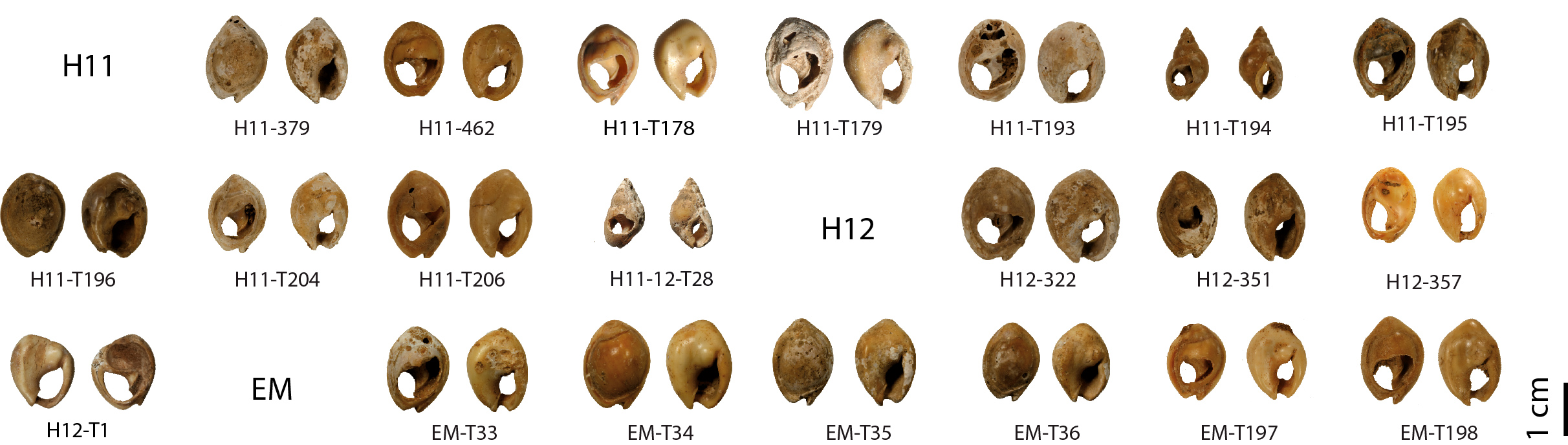

Supplement: S5 Fig — (JPG) [file pone.0338785.s005.jpg]

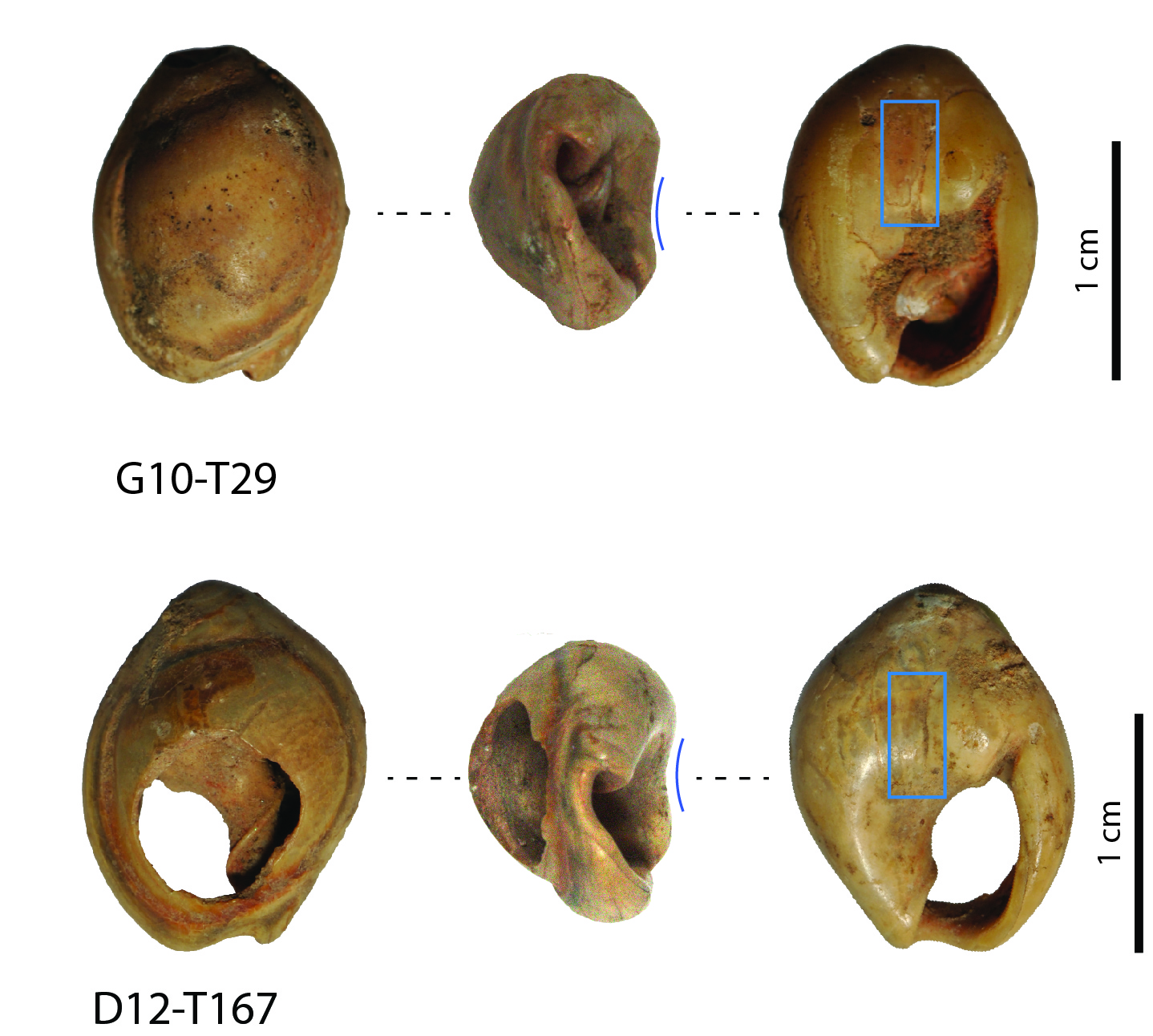

Supplement: S6 Fig — Localization of abrasions in a concave part of the ventral side. (JPG) [file pone.0338785.s006.jpg]
