# Supplementary material for: Aterian shell beads from the coastal site of El Mnasra Cave (Rabat-Témara, Morocco): Specificities of the north African MSA personal ornaments
Source: PLoS One. 2026 Mar 25;21(3):e0338785. doi: 10.1371/journal.pone.0338785 (PMC13016355; doi:10.1371/journal.pone.0338785)
Supplement: S3 Table — (DOCX) [file pone.0338785.s009.docx]

| Perforation types d'Errico et al., 2009 | a | b | c | d | e | f | g | h | i | j | k | l | m |
| --- | --- | --- | --- | --- | --- | --- | --- | --- | --- | --- | --- | --- | --- |
| Shape types (present study) | a | a | b | a | a | a | b | a | a | a | b | a | Broken Fragment |
| Perforation types (present study) | a | a | a | b | c | d | d | e | f | g | g | i | Broken Fragment |

Table SI1: Relationship between Nassariidae shell bead perforation types described by [d' Errico et al. [13](#_ENREF_13)](Figure 3), and shape and perforation types in the present study.
